# Developmentally Engineered Callus Organoid Bioassemblies Exhibit Predictive In Vivo Long Bone Healing

**DOI:** 10.1002/advs.201902295

**Published:** 2019-12-10

**Authors:** Gabriella Nilsson Hall, Luís Freitas Mendes, Charikleia Gklava, Liesbet Geris, Frank P. Luyten, Ioannis Papantoniou

**Affiliations:** ^1^ Prometheus Division of Skeletal Tissue Engineering Skeletal Biology and Engineering Research Center Department of Development and Regeneration KU Leuven O&N1, Herestraat 49, PB 813 3000 Leuven Belgium; ^2^ Prometheus Division of Skeletal Tissue Engineering KU Leuven O&N1, Herestraat 49, PB 813 3000 Leuven Belgium; ^3^ GIGA In Silico Medicine Université de Liège Avenue de l'Hôpital 11—BAT 34 4000 Liège 1 Belgium; ^4^ Biomechanics Section KU Leuven Celestijnenlaan 300C, PB 2419 3001 Leuven Belgium; ^5^ Present address: Institute of Chemical Engineering Sciences (ICE‐HT) Foundation for Research and Technology Hellas (FORTH) Stadiou St. Platani 26504 Patras Greece

**Keywords:** developmental engineering, endochondral ossification, long‐bone defect, organoid, tissue engineering

## Abstract

Clinical translation of cell‐based products is hampered by their limited predictive in vivo performance. To overcome this hurdle, engineering strategies advocate to fabricate tissue products through processes that mimic development and regeneration, a strategy applicable for the healing of large bone defects, an unmet medical need. Natural fracture healing occurs through the formation of a cartilage intermediate, termed “soft callus,” which is transformed into bone following a process that recapitulates developmental events. The main contributors to the soft callus are cells derived from the periosteum, containing potent skeletal stem cells. Herein, cells derived from human periosteum are used for the scalable production of microspheroids that are differentiated into callus organoids. The organoids attain autonomy and exhibit the capacity to form ectopic bone microorgans in vivo. This potency is linked to specific gene signatures mimicking those found in developing and healing long bones. Furthermore, callus organoids spontaneously bioassemble in vitro into large engineered tissues able to heal murine critical‐sized long bone defects. The regenerated bone exhibits similar morphological properties to those of native tibia. These callus organoids can be viewed as a living “bio‐ink” allowing bottom‐up manufacturing of multimodular tissues with complex geometric features and inbuilt quality attributes.

## Graphical abstract

Developmentally engineered callus organoids allow implementation of bone by design strategies. Callus organoids exhibit a remarkable capacity to form bone microorgans upon implantation. This capacity is linked to specific gene profiles that correspond to developmental and fracture healing processes. When assembled in vitro, the callus organoids fuse resulting in large multimodular implants able to rapidly heal critical‐sized long bone defects.

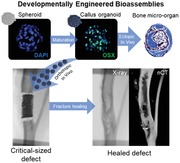

## 1 Introduction

Tissue‐engineered advanced therapy medicinal products (TE‐ATMPs) are poised to revolutionize health care by replacing or restoring the function of damaged organs. Although major advances in the field of cell therapy manufacturing have been witnessed, only a small fraction of TE‐ATMPs exhibit quality attributes that could guarantee predictive performance in vivo and hence support clinical translation.^1^, ^2^, ^3^ To tackle these hurdles, a conceptual and technical merging of developmental biology and engineering principles is taking place within regenerative medicine. These “developmental engineering” strategies strive to mimic developmental events while guaranteeing robustness and predictive outcomes in a clinical setting.^4^, ^5^, ^6^, ^7^ According to this strategy, cellular self‐assemblies and condensations of the appropriate length scale are key initiators for the formation of transient tissue structures capable of executing developmental programs with a high level of independence leading to organogenesis processes.^8^, ^9^ These processes are regulated through the activation of tissue‐specific genes and pathways characterized by a high degree of autonomy resulting in tissues that are able to undergo a similar cascade of processes even ex vivo.^10^ This type of recapitulation of developmental events has previously been demonstrated with human adult stem cells, for example, for the formation of epithelial^1^ and liver^11^ organoids.

In the context of bone tissue engineering, fracture healing of long bones includes the formation of a cartilaginous “soft callus” that subsequently is transformed into bone,^12^ a process that resembles the well‐described and tightly synchronized process of endochondral ossification in the growth plate during development.^13^, ^14^, ^15^ The autonomy of the growth plate cartilage in embryonic cartilage anlagen was previously reported, and even when the cartilage anlage was decomposed into single cells and re‐implanted subcutaneously, re‐organization occurred and a growth plate‐like structure was formed.^4^, ^10^ Furthermore, investigations inspired by “developmental engineering” demonstrated recapitulation of endochondral ossification in ectopic environments using embryonic stem cells^16^ or bone marrow mesenchymal stromal cells (BM‐MSCs)^17^, ^18^ and orthotopically using rat^19^ or human^20^ BM‐MSCs. However, only partially successful results have been demonstrated due to scalability challenges and uncontrolled complexity in 3D cell culture formats currently used for inducing chondrogenic differentiation.^18^, ^21^ The use of scaffold‐free microspheroid cultures could provide a more homogeneous 3D culture format to precisely engineer soft callus‐like microenvironments.^22^, ^23^, ^24^, ^25^ The ability to produce populations of small functional modules will constitute a major step toward the incorporation of design principles in skeletal living implant manufacturing.^2^

The formation of high‐throughput cell microspheroid populations of defined size and their use as building modules for bottom‐up tissue formation strategies is gaining momentum for various TE applications.^22^, ^26^, ^27^, ^28^ However, the construction of complex engineered tissues possessing multicomponent tissue architecture is still elusive. Although bottom‐up approaches have been suggested in recent years in order to build larger tissue structures from micromodules, the majority of these studies used cell microspheroids with minimal cell‐secreted extracellular matrix (ECM).^26^ Regarding long bone defect regeneration, there is scarce literature on the potential of modular bioengineering strategies to generate larger implants while there is no understanding yet of how this architecture dictates whole tissue function after implantation. Ideally, modules for regeneration of long bone defects should possess an autonomy that would guarantee that the repeated functional units can synergistically contribute to the regenerative process, resulting in a predictive clinical outcome.

**Figure 1:**
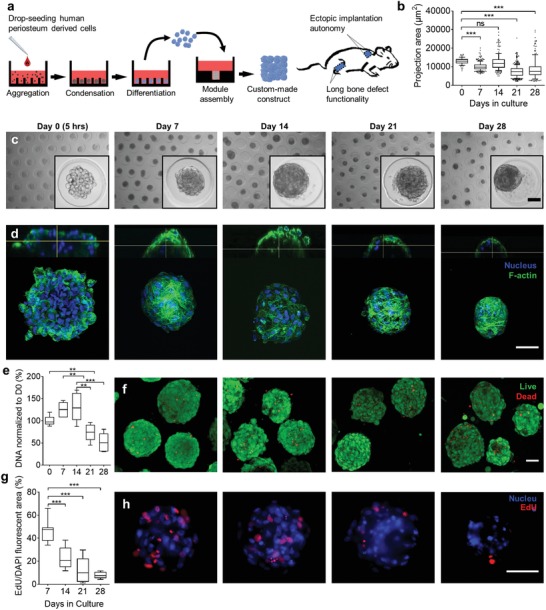
Long‐term culture of periosteal microspheroids. a) Schematic overview of the bioengineering process starting with cellular aggregation, condensation, and differentiation followed by callus organoid assembly and implantation in ectopic and orthotopic environment. b) Projection area of microspheroids over time (87–400 microspheroids, 10–90 percentiles). c) Representative bright‐field images of microspheroids over time. d) Representative 3D renderings of confocal images of stained with DAPI (nucleus) and Phalloidin (F‐actin) over time. e) DNA quantification of microspheroids over time, normalized to day 0 (5 h) (*n* = 6, 10–90 percentiles). f) Representative confocal *z*‐projection images of LIVE (green)/DEAD (red) staining over time. g) Semiquantification of cell proliferation in microspheroids over time. EdU fluorescent area was normalized to DAPI fluorescent area (10–15 microspheroids per condition, 10–90 percentiles). h) Representative fluorescent images of proliferating cells (EdU, red) in microspheroids over time, blue represents the nucleus. **p < 0.01; ***p < 0.001; one‐way analysis of variance (ANOVA) followed by Tukey's multiple comparison test. Scale bars: c,d,f,h) 50 µm.

In this work, we present a developmental bioengineering strategy based on self‐assembly of human‐periosteum‐derived cells (hPDCs). hPDCs have great promise for regeneration of long bone defects, since the majority of cells forming the “soft callus” during fracture healing are derived from the periosteum.^14^, ^15^, ^29^ In addition, recently published studies demonstrated the presence of skeletal stem cells within the periosteum with improved capacity to regenerate bone as compared to BM‐MSCs.^15^, ^30^ Herein, self‐assembly of hPDCs allowed scalable production of semiautonomous callus organoids that formed bone microorgans upon implantation. The in vitro maturation toward callus organoids was linked to gene expression patterns encountered in the embryonic growth plate and during fracture healing. Furthermore, an assembly of multiple callus organoids resulted in multimodular constructs that formed large bone organs ectopically and healed critical‐sized long bone defects in mice. In both cases, bone organs were formed in the absence of contaminating fibrotic tissue and exhibited a well‐developed bone marrow compartment, thus demonstrating the potential of this modular approach (**Figure** **1**a) for future clinical applications.

## 2 Results

### 2.1 Long‐Term Culture of Microspheroids Follows Early Pattern of Endochondral Ossification

Endochondral ossification is initiated with cell aggregation and condensation, followed by chondrocyte specification, differentiation, and formation of a cartilage tissue intermediate that subsequently is replaced by bone.^31^ Here, cell aggregation, condensation, and differentiation of hPDC microspheroids were studied over a period of 4 weeks (Figure 1b,c). The self‐aggregation process comprised two steps. Initially, over a course of 5 h (day 0), hPDCs self‐assembled to form a stack of cells until a spheroid shape was attained (Figure 1c,d; Movie S1, Supporting Information). Filamentous‐actin (F‐actin) staining demonstrated changes in the actin cytoskeleton by formation of stress fibers during the first week as well as compaction of microspheroids with a more confined cortical actin network over time and its thinning after 3 weeks (Figure 1d).

3D visualization of cell nuclei showed the presence of nuclear condensation and fragmentation indicating occurrence of apoptosis in some cells starting from day 14^32^ (Figure S1a, white arrows, Supporting Information). Furthermore, DNA quantification suggested a stable number of cells during 2 weeks followed by a 44% decrease after 3 weeks (Figure 1e). The majority of cells in the microspheroids were viable; however, an increase in dead cells was observed during the last week of the culture period (Figure 1f). Messenger ribonucleic acid (mRNA) transcripts of the marker of proliferation Ki‐67 (*MKI67*) declined after 21 days (Figure S1d, Supporting Information) and 5‐ethynyl‐2′‐deoxyuridine (EdU) staining confirmed this trend by revealing a high number of proliferating cells (46%) during the first weeks, which subsequently decreased and was almost absent after 4 weeks in culture (Figure 1g,h). This decrease in proliferation is also seen during endochondral ossification^31^ indicating chondrocyte differentiation and maturation of the microspheroid cells.

**Figure 2:**
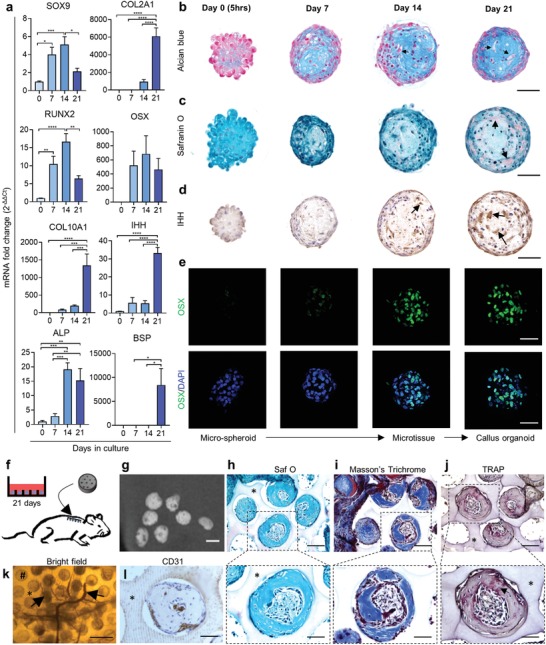
Microspheroids follow endochondral ossification patterns toward pre‐hypertrophic callus organoids able to form bone in vivo. a) Quantification of mRNA transcript of chondrogenic and pre‐hypertrophic/hypertrophic gene markers was performed and normalized to D0 (*n* = 6 mean value ± SEM). *p < 0.05; **p < 0.01; ***p < 0.001; one‐way ANOVA followed by Tukey's multiple comparison test. b–e) Representative sections of: b) Alcian Blue, c) Safranin O, d) IHH immunostaining, and e) confocal *z*‐projection image of OSX immunostaining over time. f) Schematic view of individual callus organoid implantation. g) 3D rendering of nano‐CT images after 4 weeks in vivo implantation. h) Safranin O, i) Masson's Trichrome, and j) TRAP staining after 4 weeks in vivo. k) Bright‐field image of invading blood vessels (black arrow, #: microwell) and l) CD31 immunostaining 4 weeks after implantation (* represents the agarose mold). Scale bars: b–e) 50 µm; g) 100 µm; h–j) the upper row represents 100 µm and the lower row 50 µm; k) 400 µm; and l) 50 µm.

To further define the differentiation stages of the microspheroids, gene expression of relevant markers was analyzed (**Figure** **2**a). The early chondrogenic transcription factor sex‐determining region Y box (*SOX)9* was upregulated (5‐fold) the first 14 days in culture followed by a downregulation while the cartilage matrix marker collagen type II alpha 1 (*COL2A1*) was highly upregulated (6100‐fold) after 21 days in culture. The early osteogenic and pre‐hypertrophic marker runt‐related transcription factor 2 (*RUNX2*) was upregulated after 7 (10‐fold) and 14 days (16‐fold) where after a downregulation was seen. The transcription factor osterix (*OSX* or *SP7*), which is directly regulated by *RUNX2* and expressed in pre‐hypertrophic chondrocytes and osteoblasts, followed a similar expression trend.^33^, ^34^ Distinct upregulation of the hypertrophic markers collagen type X alpha 1 chain (*COL10A1*) (1340‐fold) and Indian hedgehog signaling molecule (*IHH*) (33‐fold) was detected at day 21. In addition, alkaline phosphatase (*ALP*) gene expression was upregulated (19‐fold) at day 14 and integrin binding sialoprotein (*BSP* or *IBSP*), linked to matrix mineralization and osteoblast differentiation,^35^, ^36^, ^37^ was upregulated 8400‐fold, after 21 days in culture. No significant upregulation of the analyzed genes (*SOX9*, *COL2A1*, *RUNX2*, *OSX*, *COL10A1*, *IHH*, *ALP*, and *BSP*) was detected between day 21 and day 28 (Figure S2a, Supporting Information). Therefore, the following analyses were performed until day 21. In summary, the above results demonstrated a proliferation phase that was interchanged with cellular differentiation and maturation defined by genes associated with both hypertrophic chondrocyte and osteogenic differentiation.

### 2.2 Microspheroids Mature toward Pre‐Hypertrophic Callus Organoids That Form Bone Microorgans In Vivo

The gene expression analysis indicated chondrogenic differentiation toward hypertrophy in combination with osteogenic differentiation at day 21 (Figure 2a). Furthermore, Alcian Blue staining at low pH, specific for glycosaminoglycan (GAG), confirmed an increased presence of cartilage‐like ECM within the microspheroids, and pre‐hypertrophic like cells were visible after 3 weeks in culture (Figure 2b, black arrows). Safranin O staining demonstrated slight presence of cartilage‐specific sulfated GAGs after 21 days in culture (Figure 2c) and immunostaining confirmed the presence of IHH, OSX, and COL2 protein after 14 days in culture (Figure 2d,e; Figure S1b,d,e, Supporting Information). The gene expression and histological analysis demonstrated that the microspheroids, containing ≈250 aggregated cells (day 0, Figure 2b,c), matured into microtissues with differentiated cells and ECM (day 14, Figure 2b,c).

Based on the upregulation of hypertrophic gene markers (Figure 2a) and the presence of pre‐hypertrophic cells (Figure 2b), day 21 microtissues were chosen to be implanted subcutaneously to evaluate their capacity to mature into bone in vivo. Implantation of whole agarose microwell platforms with a diameter of 5 mm was carried out in immunodeficient mice to ensure that microtissues would remain entrapped in their microwells (Figure 2f). After 4 weeks of ectopic implantation, nano‐computed tomography (nano‐CT) scans demonstrated the formation of distinct mineralized spheres (Figure 2g) with a volume of (5.4 ± 3.54) × 10^5^ µm^3^ and an average diameter of 209 ± 41 µm (24 spheres quantified from three explants). Histological sections further demonstrated the presence of bone matrix (Figure 2h,i) surrounding a marrow compartment with osteoclast activity (Figure 2j) and blood vessels (Figure 2k,l). These data demonstrated the development of microtissues with proteoglycan‐rich ECM positive for IHH and COL2. Furthermore, these microtissues were able to form bone microorgans in vivo (Figure 2g), confirming that these implants behaved as single semiautonomous bone‐forming modules in vivo acting as callus organoids. This defines a maturation process from microspheroids (day 0) to microtissues (day 14) and finally callus organoids (day 21) (Figure 2e).

### 2.3 Callus Organoids Fuse into Larger Constructs In Vitro

**Figure 3:**
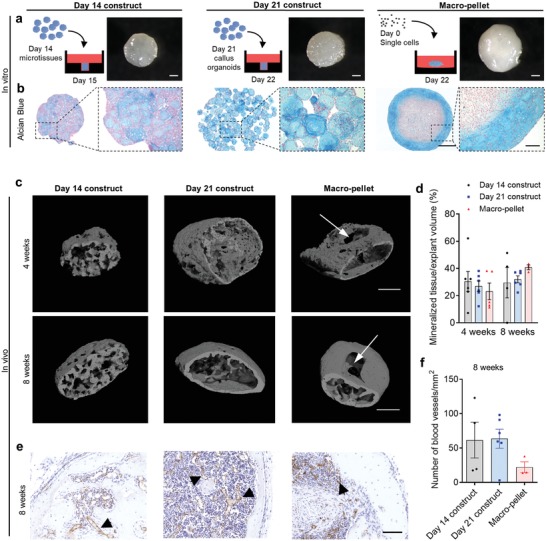
Assembly of cartilage intermediate microtissues into larger bone forming constructs. a) Schematic drawing demonstrating module assembly into an agarose macrowell (left) and representative photographs of the day 14, day 21 constructs, and Macropellet (right). b) Alcian Blue staining of fused constructs and Macropellet. c) 3D rendering of nano‐CT images 4 and 8 weeks after implantation. d) Quantification of mineralized tissue 4 and 8 weeks after implantation (mean value ± SEM, *n* = 3–6). e) Representative images of CD31 immunostaining (black arrows demonstrate blood vessels), and f) quantification 8 weeks after implantation (mean value ± SEM, *n* = 3–6). ANOVA followed by Tukey's multiple comparison test. Scale bars: a) 500 µm, b) 500 and 100 µm, c) 500 µm, and d) 100 µm.

In order to demonstrate that the above‐mentioned microtissues and callus organoids can be used as building modules to form larger constructs, we initially studied the fusion process of two callus organoids. Despite long‐term culture as microtissues, creating a substantial amount of secreted ECM, the callus organoids spontaneously fused over 24 h (Movie S2, Supporting Information). Subsequently, ≈3000 modules were flushed out of their microwells and assembled in an agarose well (2 mm diameter and depth) for fusion into a multimodule construct (**Figure** **3**a; Figure S2b, Supporting Information). 14 (microtissues) and 21 day (callus organoids) modules were chosen for further analysis based on chondrogenic (*SOX9* and *COL2A1*) and hypertrophic (*COL10A1*, *IHH*, and *ALP*) gene markers (Figure 2a,d,e), as well as cell morphology (Figure 2b–d). Both 14 day and 21 day modules fused into larger constructs that could be handled and transported (Figure 3a); however, single callus organoid structures were still visually discernible in 21 day constructs. As a control to these structures, a macropellet formed with the same number of cells and cultured for 3 weeks in the same media formulation was introduced (Figure 3a, Macropellet).

Alcian Blue staining demonstrated increased module fusion within the day 14 constructs as compared to day 21 constructs (Figure 3b), albeit both module constructs contained positive staining thoughout their structures. In contrast, the macropellet, not assembled with modules, did only show Alcian Blue staining at the periphery (Figure 3b). Safranin O staining corresponded to the Alcian Blue staining seen in macropellets. In contrast, Safranin O positive areas were found throughout the day 21 constructs (Figure S3a, Supporting Information). None of the constructs demonstrated positive staining for Alizarin red or von Kossa (Figure S3b,c, Supporting Information), indicating that mineralization was not present in the constructs. In conclusion, these results demonstrated the formation of larger constructs through assembly of micromodules resulting in more homogenously distributed GAG‐rich ECM as compared to macropellets (Figure 3b).

### 2.4 Assembled Callus Organoids Form Single Large Bone Organs In Vivo

Next, day 7, 14, and 21 constructs, as well as the macropellets were implanted ectopically in immunodeficient mice to investigate their capacity to form bone in vivo (4 and 8 weeks). None of the day 7 constructs were retrieved (*n* = 4). However, mineralization was detected with nano‐CT in the other three conditions after 4 week implantation (Figure 3c). No significant difference in mineralization percentage was seen between the conditions after 4 or 8 weeks. However, a nonmineralized core was detected in the macropellet at both timepoints (Figure 3c, white arrows). Furthermore, after 8 weeks' ectopic implantation, the day 21 constructs and macropellets contained a mineralized cortex, while the mineralized tissue in the day 14 constructs appeared porous hence less mature. The number of blood vessels was quantified with CD31 immunostaining, and no significant difference between the constructs was detected although a larger number of day 21 constructs (5/6) contained a high amount (>50 blood vessels mm^−2^) of blood vessels as compared to day 14 constructs (2/4) and macropellets (0/3) (Figure 3e,f; Figure S3d, Supporting Information).

**Figure 4:**
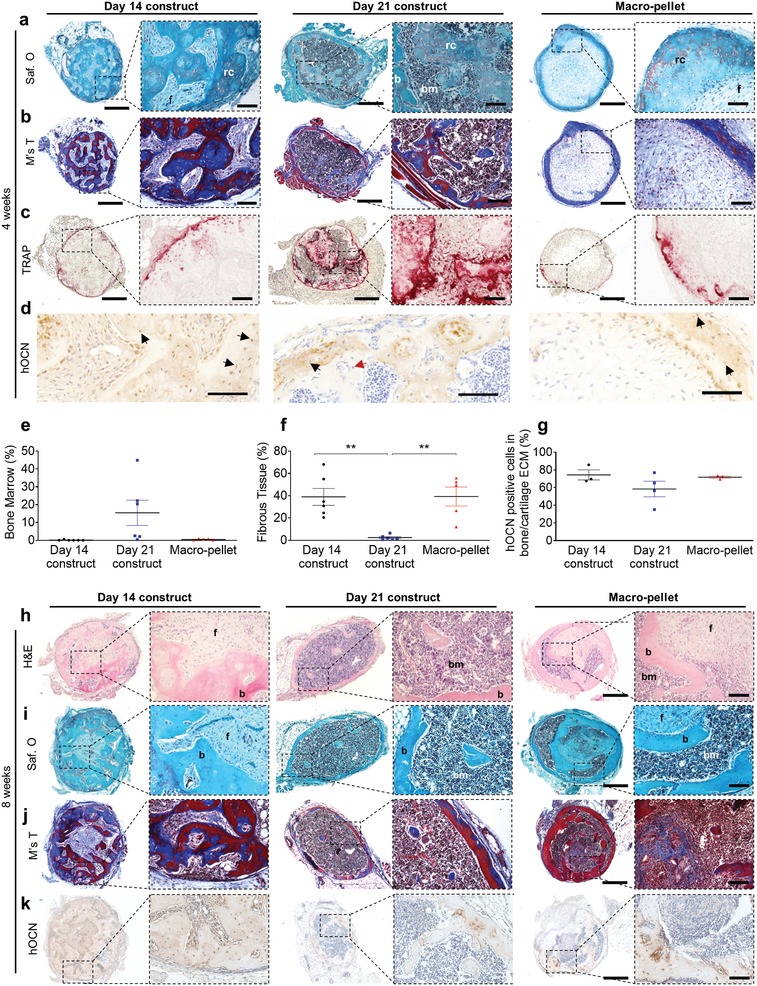
Histological assessment after ectopic implantation. a) Safranin O, b) Masson's Trichrome, c) TRAP, and d) hOCN staining of day14, day 21 constructs, and Macropellet 4 weeks after in vivo implantation. e–g) Quantification (mean value ± SEM) of: e) bone marrow compartment (*n* = 5–6), f) fibrous tissue area (*n* = 5–6), and g) hOCN positive cells (*n* = 3–4) 4 weeks after implantation. h) Hematoxylin and eosin (H&E), i) Safranin O, j) Masson's Trichrome (M's T), and k) hOCN staining after 8 weeks in vivo implantation. *p < 0.05; **p < 0.01; ***p < 0.001; one‐way ANOVA followed by Tukey's multiple comparison test. Scale bars: a–c,h–j) 500 µm (left) and 100 µm (right) and d) 100 µm.

Safranin O and Massons's Trichrome staining on histology sections after 4 weeks' implantation revealed that day 21 constructs contained bone, bone marrow, as well as remodeling cartilage indicating the occurrence of endochondral ossification (**Figure** **4**a; Figure S3f, Supporting Information). Although no significant difference was detected, limited bone marrow compartments were seen in the day 14 constructs and macropellets in contrast to the day 21 constructs (Figure 4a,b,e). Strikingly, significant areas of fibrotic tissue were detected in both day 14 constructs and macropellets as compared to day 21 constructs (Figure 4f). Furthermore, tartrate‐resistant acid phosphatase (TRAP) staining (Figure 4c) demonstrated osteoclast activity in all constructs although more prominent in day 21 constructs. Human osteocalcin (hOCN) staining demonstrated that implanted cells contributed to the bone formation in all constructs (day 14 construct: 74 ± 10%, day 21 construct: 58 ± 18%, and macropellet: 72 ± 2%) (Figure 4d,g; Figure S3e, Supporting Information).

Furthermore, hematoxylin–eosin (H&E), safranin O, and Masson's Trichrome staining revealed mature bone in all conditions after 8 weeks' implantation, but the day 14 constructs and macropellets still contained domains of fibrotic tissue which were absent in the day 21 constructs (Figure 4h–j). In addition, OCN positive cells of human origin were present also after 8 weeks' implantation (Figure 4k). Although mineralized, some of the day 21 constructs maintained a hypertrophic chondrocyte phenotype after both four (three of six implants) and eight (one of six implants) weeks in vivo implantation (Figure S3f, Supporting Information). Taken together, these results supported that callus organoids fused into larger day 21 constructs in vitro and further developed into bone organs in vivo.

### 2.5 Temporal Gene Expression Patterns during Callus Organoid Formation Follow the Endochondral Ossification Process toward a Niche for Matrix Remodeling and Bone Organ Formation

**Figure 5:**
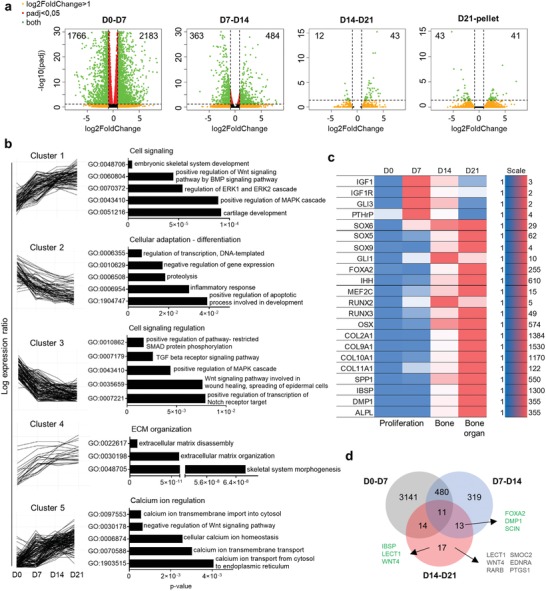
RNA sequencing analyses of spheroids and Macropellet (*n* = 3–4). a) Volcano plots of differentially expressed genes from RNA‐seq data between the different spheroids and Macropellet. *X*‐ and *Y*‐axes represent log_2_fold change and log_10_ *p‐*value, respectively, and green dots represent genes with log2FoldChange > 1 and padj < 0.05. b) Gene Ontology (GO) Biological Processes (2017b) of genes within the five different clusters of the 400 most variable genes (log2FoldChange > 1 and padj < 0.05). c) Heat map of spheroid mRNA transcript levels of genes regulating endochondral ossification (relative expression value for each gene). d) Venn diagram of the number of significant differentially expressed genes (green dots in (a)) for the different spheroid maturations. Green text represents genes associated with endochondral ossification and gray text genes associated with angiogenesis.

To better explain the differentiation pathway of the callus organoids, an RNA sequencing analysis of D0 (5 h), D7, D14, and D21 modules was performed demonstrating a similar trend as the limited gene expression analysis (Figure 2a; Figure S4a,b Supporting Information). Furthermore, the number of significant (*p* < 0.05 and log_2_‐fold > 1) differentially expressed genes decreases over time from 3949 (D0–D7) and 847 (D7–D14) to 55 (D14–D21) and 84 (D21–pellet) (**Figure** **5**a) indicating that the most dramatic changes occurred at the early stages of differentiation.

Pathway analysis using Enrichr^38^, ^39^ with WikiPathway (2016) grouped upregulated genes (D0–D21) into endochondral ossification (WP474, adj. *p*‐value 1.0e‐13) and embryonic skeletal system development (Gene Ontology (GO): 00 48706, adj. *p*‐value 1.379e‐8) from GO Biological Process (BP) (Data File S1, Supporting Information).^38^, ^39^, ^40^ Next, unsupervised clustering was performed on the 400 most variable genes in order to gain a holistic overview of signaling action during the callus organoid maturation process and the GO enrichment for each cluster was defined (Data Files S2 and S3, Supporting Information). The first cluster with a continuously upgoing trend included genes enriched to skeletal and cartilage development and regulation of mitogen‐activated protein kinases (MAPK) and ERK1/2 signaling (where ERK = extracellular signal‐regulated kinase) involving the wingless‐INT (WNT), bone morphogenetic protein (BMP), and fibroblast growth factor (FGF) signaling (Figure 5b). These are crucial signaling pathways driving endochondral ossification working in a converging manner toward chondrocyte hypertrophy.^41^ Interestingly, genes related to WNT signaling were also present in the transient downregulated cluster 3. This cluster included genes related to transforming growth factor beta (TGF‐β)/BMP related SMAD and WNT signaling motivating a converging crosstalk during callus organoid maturation. In addition, Notch, which is important for stem cell maintenance, suppression of chondrocyte differentiation, and proliferation,^42^ was represented in the downregulated cluster 3. These two clusters (clusters 1 and 3) indicate cell signaling regulation analogous to the molecular cascade of events present during endochondral ossification.^41^

Cluster 2, with constant downregulation, included genes associated with DNA transcriptional activity correlating with the decrease in cell proliferation occurring during the transition from proliferative to hypertrophic chondrocytes^43^ which was indicative also in our data (Figure 1g,h). Genes associated with ECM disassembly (*MMP13*) and produced by hypertrophic chondrocytes (*COL10A, COL9*, and *SPP1*) were grouped in the constantly upregulated cluster 4 supporting maturation toward hypertrophic callus organoids that exhibited a high turnover and capacity to remodel the surrounding matrix. In addition, genes linked to calcium‐ion regulation were highly represented in the upregulated cluster 5, suggesting a gradual transition to a pre‐hypertrophic niche favoring mineralization, although no in vitro mineralization was detected (Figure S3b,c, Supporting Information).^44^

The GO enrichment of the unsupervised clusters demonstrated that the callus organoid maturation followed signaling pathways regulating endochondral ossification. This was further supported by analysis of well‐known regulators. During the first phase (D7), important regulators of chondrocyte proliferation, differentiation, and organization were upregulated, including *IGF1* and its receptor *IGF1R*,^45^ *GLI3*,^46^, ^47^, ^48^ *PTHrP*,^49^ and the SOX trio (*SOX 5*/*6*/*9*)^50^ (Figure 5c). From day 14 onward, the *PTHrP* positive state converted into a *IHH* positive state, followed by increased expression of chondrocyte hypertrophy activators, such as *GLI1*, *FOXA2*, *MEF2C*, *OSX, RUNX2*, and *RUNX3* 48, 51 (Figure 5c). This pattern was mirrored in the gene expression of matrix proteins and regulators involved in endochondral ossification with a distinct upregulation of collagens (*COL2A1, COL9A1, COL10A1*, and *COL11A1*) and signaling factors correlated to pre‐hypertrophic/hypertrophic chondrocytes and osteoblasts (*SPP1, IBSP, DMP1*, and *ALPL*).^48^, ^51^

These data demonstrate a regulatory “switch” between D7 and D14 plausibly crucial for bone formation in vivo. Genes significantly upregulated between both D7–D14 and D14–D21 included *FOXA2*, *DMP1*, and *SCIN* which are crucial for chondrocyte hypertrophy,^52^ cartilage–bone transition,^53^ and bone resorption,^54^ respectively, indicating their significant role in bone formation and also in the current manufacturing approach (Figure 5d). Subsequently, comparison from D14 to D21 indicated further maturation linking to the in vivo formation of a bone organ with matrix remodeling and the presence of bone marrow (Figure 4). Since only a limited number of genes were significantly changed (55 up/downregulated genes, Figure 5a) during this period, individual analysis was performed for these genes (Figure S4c, Supporting Information). Around 15 of the 43 differentially upregulated genes (Figure S4c, Supporting Information) have been associated with endochondral ossification and pre‐hypertrophic/hypertrophic chondrocytes (Table S1, Supporting Information) whereof *IBSP*,^36^, ^37^ chondromodulin (*CNMD* or *LECT1*),^55^, ^56^ and *WNT4* 57 were exclusively significant for D14–D21 (Figure 5d). Interestingly, 15 of the 55 significantly changed genes D14–D21 (Figure S4c, Supporting Information) have been associated with regulation of angiogenesis (Table S2, Supporting Information), a pivotal event during the transition from cartilage to bone in endochondral ossification. Of these angiogenic genes, six were exclusively differentially expressed between D14 and D21 (Figure 5d). Conclusively, the RNA‐seq analysis demonstrated endochondral maturation from initial microspheroids (aggregated cells) to callus organoids (cells and ECM) exhibiting pre‐hypertrophic characteristics and active remodeling of the secreted ECM resulting in bone organ formation in vivo.

### 2.6 Assembled Callus Organoids Heal Critical‐Sized Long Bone Defects

**Figure 6:**
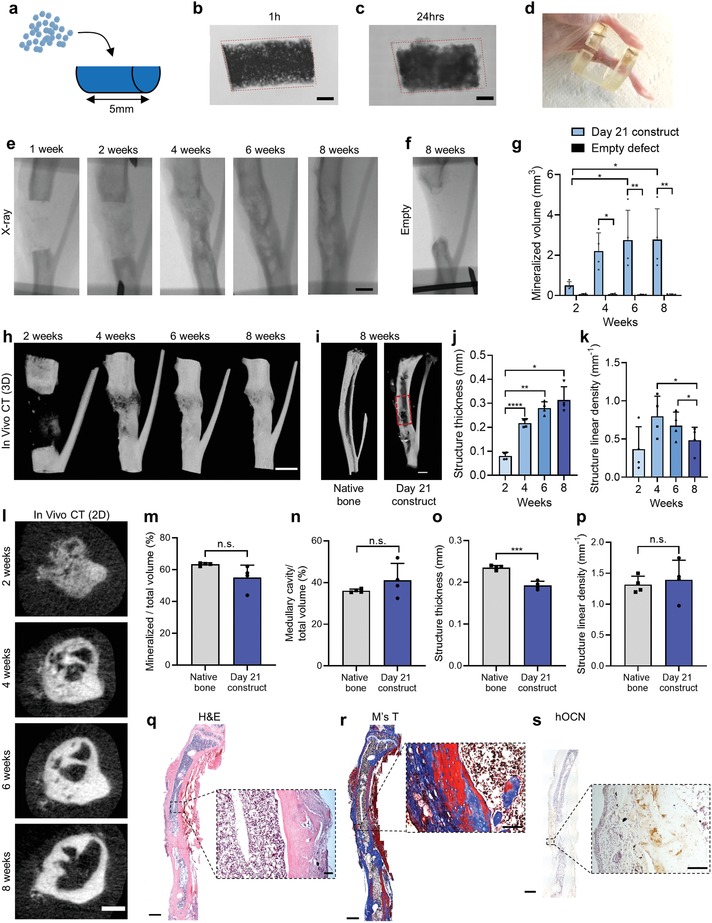
Healing of murine critical‐sized long bone defect. a) Schematic visualization of implant formation. b,c) Bright‐field image 1 h (b) and 24 h (c) after callus organoid assembly. d) Photograph of a 4 mm tibia defect after healing. e) X‐ray images of tibia defect with a day 21 construct. f) Negative control: X‐ray of empty defect after 8 weeks. g) Quantification of mineralized volume in defects with day 21 construct and empty defects (*n* = 4 animals for each condition, two‐way ANOVA followed by Tukey's multiple comparison test). h) Nano‐CT 3D rendering images over time of defect with day 21 construct. i) Cross section of 3D rendering of native tibia and defect 8 weeks after day 21 construct implantation. j–l) In vivo CT quantification of structure: j) thickness, and k) linear density over time visualized with l) in vivo CT 2D images. m–p) Comparison between native tibia and healed defect 8 weeks after construct implantation was demonstrated by ex vivo nano‐CT quantification of: m) mineralized tissue (%), n) medullary cavity (%), o) structure thickness, and p) structure linear density (*n* = 4, unpaired *t*‐test). q) H&E, r) Masson's Trichrome, and s) hOCN immunohistological staining of defect 8 weeks after day 21 construct implantation. *p < 0.05; **p < 0.01; ****p < 0.0001. Scale bars: b,c,e,h,i) 1 mm, l) 500 µm, q–s) overview 1 mm and zoom‐in 100 µm.

Based on the ectopic implantation and RNA sequencing results, day 21 modules were defined as “callus organoids” and selected as modules for the formation of larger constructs and orthotopic implantation in a murine, critical‐sized long bone defect.^58^ An agarose mold based on the dimensions of the critical‐sized defect and with the decrease in size during fusion of callus organoids accounted for was fabricated (**Figure** **6**a). Next, ≈6000 callus organoids were seeded into the agarose mold (Figure 6b) and fused during 24 h resulting in a construct (≈4.5 mm length and 2 mm wide) (Figure 6c) that was fitted into the tibia defects of immunodeficient mice (Figure 6d).

X‐ray images and 3D renderings of in vivo CT scans demonstrated occurrence of mineralization after 2 weeks, and bridging of defects was detected after 4 weeks followed by increased corticalization until week 8 (Figure 6e,h; Figure S5a, Supporting Information). No bridging was detected in the empty defects after 8 weeks (Figure 6f) while quantification of in vivo CT images confirmed the increase in mineralized tissue over time in experimental conditions (Figure 6g). Cross section of the nano‐CT 3D rendering from week 8 demonstrated the presence of cortical bone in the defect with a nonmineralized compartment in the center, suggesting a defined bone marrow cavity (Figure 6i). Structure thickness increased significantly from week 2 to 4 correlating to the time of defect bridging (Figure 6j; Figure S5a, Supporting Information). Furthermore, the number of structures decreased from week 4 to 8 indicating remodeling from a trabecular to a more cortical structure, which was also visible on in vivo CT images (Figure 6k,l).

Next, the healed defects at week 8 were compared to native tibia at the same location as the defect, in mice of the same age and gender. No significant differences were found regarding mineralized percentage, volume (Figure 6m; Figure S5b, Supporting Information), structure linear density (Figure 6p), or medullary cavity occupancy (Figure 6n), while the medullary volume in healed defects was significantly larger than in native bone (Figure S5c, Supporting Information). In addition, structure thickness was lower in the healed defects as compared to native bone indicating that longer healing time may be necessary for full regeneration (Figure 6o).

H&E and Masson's Trichrome staining after 8 weeks confirmed full bridging (3/4) with the presence of mature bone and bone marrow (Figure 6q,r; Figure S5d,e, Supporting Information), and hOCN staining (Figure 6s) revealed the contribution of donor cells to the bone formation process. In conclusion, the assembly of multiple callus organoids into an easy‐to‐handle scaffold‐free implant resulted in full bridging of a critical‐sized long bone defect by the formation of cortical‐like bone tissue with a medullary cavity containing bone marrow with the absence of fibrous tissue. In addition, structural characterization of the regenerated defect showed high similarities to native tibia.

## 3 Discussion

In this work, we developed a bottom‐up modular strategy for scalable biofabrication of cartilage intermediate tissues that were able to form ossicles without contaminating tissue compartments while exhibiting a unique capacity to heal critical‐sized long bone defects. During native fracture healing, cells from the periosteum are the main contributors of the callus.^14^, ^29^ These cells have recently been shown to possess a higher regenerative capacity than bone marrow mesenchymal cells and contain a skeletal stem cell population with distinct functions during endogenous bone repair.^15^, ^30^ Moreover, it was recently reported that periosteum contains not only renewable skeletal stem cells forming membranous, cortical bone, but also endochondral bone upon damage.^59^ Hence, this understudied progenitor cell source possesses critical advantages in terms of clinical application for the design of engineered ATMPs aiming to heal large long bone defects.

To date, the formation of cartilage intermediates in vitro was obtained through the use of pellets containing large amount of cells (>2 × 10^5^ cells).^17^, ^18^, ^60^, ^61^ However, the use of such methods has resulted in diffusion‐related challenges such as the formation of undifferentiated tissue compartments in vitro which hinder the concerted progression of tissue maturation to their final phenotype upon implantation.^18^ This was also detected in our study by the large fibrotic compartments encountered within macropellet explants (Figure 3). In addition, when chondrogenic^61^ and hypertrophic^62^ pellets were fused into larger structures, limited remodeling in vivo was shown. Hence, we designed cell microspheroids (comprised of 250 cells) that would not exceed 150 µm in diameter to match with the length scale that diffusible signals can be transported and to mimic the initial developmental event of growth plate formation (condensation) whereby only a few hundred cells are needed.^63^ During differentiation, we observed that cells underwent a cascade of molecular and cellular events that reflect endochondral ossification allowing them to transform from cellular spheroids to semiautonomous microtissue structures, callus organoids, capable of undergoing organogenesis (Figures 1, 2, 3). In addition, the assembly of callus organoids into larger tissue structures resulted in implants containing active regenerative components throughout their structure. Ultimately, the populations of callus organoids described in our study could be viewed as a living “bio‐ink” that also allows the formation of scaffold‐free tissue structures with intricate geometric features (Figure S6, Supporting Information).^64^

In order to obtain quality attributes that could be linked to the functionality of the callus organoids, high‐depth transcriptomic profiling was carried out. Sets of genes were determined to provide signatures to identify whether engineered microtissue niches have attained the degree of autonomy required for bone organ formation. These were compared to recent studies focused on the identification of transcription factor panels that control differentiation transitions from one zone to the other in the growth plate.^48^, ^51^ With this comparison, we were able to discern similar temporal gene regulation kinetics and link the phenotypic state and semiautonomous function of our microtissues to that of an “early pre‐hypertrophic” stage for day 14 modules (microtissues) and that of “late pre‐hypertrophic” stage for day 21 modules (callus organoids). In addition, GO term analysis of the 400 most variable genes revealed additional etiologies (Figure 5b) for the striking bone organ formation observed in our study. Upregulated clusters indicated gradual transition to pre‐hypertrophy, favoring mineralization as well as ECM disassembly and organization (Figure 5b). Apart from the relevance of ECM disassembly and organization in the transition from hypertrophic cartilage to bone,^65^ this property could also be key in regulating the orchestrated transition of the multimodular constructs into a single ossicle by facilitating ECM reorganization and vascular invasion across the implant. This could also explain the rapid vascularization and bone marrow formation of day 21 constructs observed as early as 4 week postimplantation (Figure 4) as well as host integration in the long bone defect (Figure 6). Chan et al.^66^ have previously demonstrated the importance of endochondral ossification for the formation of hematopoietic stem‐cell (HSC) niches, and here we provide a set of metrics that would allow fine tuning and robust bone organ formation.

Although scaffold‐free constructs are beneficial for mimicking native tissue morphology, a combination of the callus organoids with suitable biomaterials could further enable upscaling into centimeter‐sized implants and even enhance their performance.^67^, ^68^, ^69^ Functionalized biomaterials possessing molecular signatures relevant to the timescales of the differentiation cascades and the proper length scale could interact and support endochondral ossification events. Petersen et al. recently demonstrated that the architecture of collagen scaffolds can direct endochondral fracture healing in vivo.^70^ They showed that scaffold pores oriented along the defect resulted in ECM alignment and controlled invasion of progenitor cells and blood vessels leading to the onset of endochondral ossification. In the present work, we observed rapid vascularization and bone formation which was attributed to active ECM remodeling, a dynamic property that could further be supported by properly designed scaffolds through the delivery of relevant enzymes.^71^

Localized delivery of growth factors through tailored biomaterials could further direct tissue maturation in vivo while avoiding release of supraphysiological levels, which for BMP‐2 has been proven to cause severe side effects including swelling and heterotopic bone formation.^72^ Herberg et al. demonstrated that a combination of BMP‐2 and TGF‐β1 releasing microparticles in cell‐based constructs resulted in mineralized bridging in tibia defects which was further enhanced by mechanical stimulation of the defect.^73^ Furthermore, nanoscale fibronectin coatings on polycaprolactone scaffolds were shown to allow incorporation of ultralow dose BMP‐2 (100 ± 8 ng cm^−2^) resulting in bone formation in vivo.^74^ However, it is of note that the use of biomaterials could also have adverse effects for tissue regeneration when their properties are not coupled to the precise regenerative context. For example, collagen I scaffolds used in both clinical and research applications for bone regeneration were recently shown to impede osteogenic differentiation and fracture healing.^75^ This highlights the importance of thorough understanding of the interaction between the scaffold material and the biology for a specific application.

There are still a number of technical challenges that need to be addressed for future biomanufacturing of callus organoids for mass production. The transition of the static process developed in this study to bioreactor systems where thousands of organoids could be generated could aid in its full automation and enhance its capability. In addition, the transfer of this process to stirred bioreactor systems could potentially allow increased flexibility in terms of achievable scale.^76^ At the same time, already available technologies for isolating single microtissue modules for at‐line quality controls could provide an ideal method for real‐time evaluation of their degree of autonomy^77^ allowing the implementation of real‐time potency monitoring as envisaged in the quality by design paradigm for cell therapy. Bioprinting technologies with the capacity to manipulate single spheroids have been developed through laser‐induced forward transfer, a high‐resolution method using laser pulses.^78^ Finally, robotic devices have been shown to possess the capacity to manipulate single spheroids and positioning them in preordered grids allowing them to fuse^79^, ^80^ or depositing them in printed scaffolds.^78^

Another technical bottleneck that will need to be addressed is the vascularization of multicentimeter‐sized implants. Although chondrocytes possess resistance to stress conditions found at the implantation site such as hypoxia and low nutrient availability, it is expected that vascularization will be a prerequisite for cell survival in large implants.^81^ Recently, vascularized structures based on the concomitant use of mesenchymal condensations, of similar dimensions to the ones presented here and endothelial cells, exhibited improved in vivo functionality.^8^ Moreover, sacrificial writing into functional tissue (SWIFT) bioprinting with direct fabrication of vasculature in organoid suspensions could also be employed for introducing vasculature patterns when upscaling to larger callus‐organoid‐based implants.^82^ In addition, using purified stem cell populations recently described by Chan et al.^83^ could substantially enhance the potential and efficiency of the strategy described in this work.

## 4 Conclusion

In conclusion, the described callus organoids provide an engineering approach for predictive design of large‐scale living implants. The callus organoids exhibited a deterministic behavior by reaching autonomy thresholds attributed to synchronized activation of molecular pathways providing robustness and potentially facilitating regulatory approval and safety. Furthermore, this process is scalable both in terms of production of single callus organoids and in terms of tissue implant size and at the same time allowing the design of intricate geometric features. Importantly, the in vivo functional assessment of orthotopic bone formation with bridging of the long bone defect took place within the timelines of natural fracture healing and resulted in a bone structure highly resembling native long bone.^12^ With these advancements, we believe that future biofabrication of skeletal implants using callus organoids will follow design principles resulting in achieving “bone by design”. This will eventually pave the way for the biomanufacturing of clinically relevant implants possessing robust functionality and causal connection with the clinical outcome. This can revolutionize the mitigation of currently unmet clinical challenges such as healing of critical‐size long bone defects.

## 5 Experimental Section

*Cell Expansion*: hPDCs were isolated from periosteal biopsies of nine different donors, and two different cell pools were created (ages of 29 ± 12 and 14 ± 3 years) as previously described.^84^ The hPDC pools were expanded (5700 cells cm^−2^) until passage 7 (in vivo, RNA‐seq) and 10 (in vitro) at 37 °C, 5% CO_2_, and 95% humidity in Dulbecco's modified Eagle medium (DMEM, Life Technologies, UK) with 10% fetal bovine serum (HyClone FBS, Thermo Scientific, USA), 1% antibiotic–antimycotic (100 units mL^−1^ penicillin, 100 mg mL^−1^ streptomycin, and 0.25 mg mL^−1^ amphotericin B), and 1 × 10^−3^ m sodium pyruvate (Life Technologies, UK). Medium was changed every 2–3 days, and cells were harvested with TrypLE Express (Life Technologies, UK) at a confluence of 80–90%. TrypLE Express was used for all passaging and harvesting steps during cell handling. The ethical committee for Human Medical Research (Katholieke Universiteit Leuven) approved all procedures, and patients' informed consent forms were obtained (ML7861).

*Formation of Microspheroids*: Agarose microwell inserts for formation of a high number of microspheroids with homogeneous size distribution were created as previously described by Leijten et al.^85^ Briefly, 3 % (w/v) Agarose (Invitrogen, Belgium) was poured onto a polydimethylsiloxaan (PDMS, Dow Corning Sylgard 184 elastomer, MAVOM Chemical Solutions) master mould containing pillars with a diameter of 200 µm. The agarose was let to solidify where after microwell inserts with an area of ≈1.8 cm^2^ were punched out, placed in 24‐well plates, 1 mL of phosphate‐buffered saline (PBS; Lonza, Verviers, Belgium) was added and the wells were sterilized under UV for 30 min. Each well insert contained ≈2000 microwells. hPDCs were harvested and seeded with a concentration of 500 000 cells per well to obtain ≈250 cells per spheroid after self‐aggregation. Microspheroids were differentiated into microtissues in a serum‐free chemically defined chondrogenic medium (CM) containing LG‐DMEM (Gibco) supplemented with 1% antibiotic–antimycotic (100 units mL^−1^ penicillin, 100 mg mL^−1^ streptomycin, and 0.25 mg mL^−1^ amphotericin B), 1 × 10^−3^ m ascorbate‐2 phosphate, 100 × 10^−9^ m dexamethasone, 40 µg mL^−1^ proline, 20 × 10^−6^ m of Rho‐kinase inhibitor Y27632 (Axon Medchem), ITS+ Premix Universal Culture Supplement (Corning) (including 6.25 µg mL^−1^ insulin, 6.25 µg mL^−1^ transferrin, 6.25 µg mL^−1^ selenious acid, 1.25 µg mL^−1^ bovine serum albumin (BSA), and 5.35 µg mL^−1^ linoleic acid), 100 ng mL^−1^ BMP‐2 (INDUCTOS), 100 ng mL^−1^ growth/differentiation factor 5 (GDF5) (PeproTech), 10 ng mL^−1^ TGF‐β1 (PeproTech), 1 ng mL^−1^ BMP‐6 (PeproTech), and 0.2 ng mL^−1^ basic FGF‐2 (R&D systems).^86^ Half of the media volume was changed every 3–4 days.

*Viability Assay*: Cell viability in microspheroids was assessed qualitatively with LIVE/DEAD Viability/Cytotoxicity Kit (Invitrogen, USA) for mammalian cells by following the manufacturer's protocol. Briefly, microspheroids were rinsed with PBS, where after they were incubated in 2 × 10^−6^ m Calcein AM and 4 × 10^−6^ m ethidium homodimer‐1 for 30 min at 37 °C, 5% CO_2_, and 95% humidity. Stained microspheroids were visualized with a confocal microscope ZEISS LSM 510 META (Cell imaging core facility of KU Leuven) with 4 µm thick slices.

*Cell Proliferation Assay*: Cell proliferation during microspheroid differentiation was visualized with Click‐iT EdU Imaging Kit (Life Technologies, USA) according to the manufacturer's protocol. Briefly, 10 × 10^−6^ m EdU was added to the microspheroids during 4 days for each time point. Next, samples were fixed in 4% paraformaldehyde (PFA), EdU was detected with Alexa Fluor azide, stained with Hoechst 33 342 (5 µg mL^−1^) followed by visualization with a Leica M165 FC microscope (Microsystems, Belgium). The percentage of EdU/Hoechst (proliferating per all cells) stained area was quantified using ImageJ software^87^ for 10–15 microspheroids per time point.

*Cytoskeleton and Nuclei Visualization*: Cell nucleus and F‐actin distribution within microspheroids was visualized by staining with 2.5 µg mL^−1^ 4′,6‐diamidino‐2‐phenylindole (DAPI) (Invitrogen) and 0.8 U mL^−1^ Alexa Fluor 488 phalloidin (Invitrogen) during 1 h at room temperature. Stained spheroids were imaged with an inverted laser scanning fluorescence confocal microscope ZEISS LSM 510 META (Cell imaging core facility of KU Leuven) with 1 µm thick slices using an argon ion 488 nm and MaiTai laser.

*DNA Quantification, Total RNA Extraction, and Quantitative Reverse Transcription–Polymerase Chain Reaction Analysis*: Quantitative real‐time polymerase chain reaction (qRT‐PCR) was used to quantify mRNA of markers relevant for endochondral ossification. Pooled microspheroids (≈2000 microspheroids represent *n* = 1) were washed in PBS followed by cell lysis in 350 µL RLT lysis buffer (Qiagen, Germany) and 3.5 µL β‐mercaptoethanol (Sigma Aldrich, Germany), vortexed and stored at −80 °C. DNA assay kit QuantiT dsDNA HS kit (Invitrogen) was used to quantify the DNA content for each condition. Cell lysate was spun down and the DNA assay was performed according to the manufacturer's protocol. RNeasy Mini Kit (Qiagen) was used to isolate the total amount of RNA from lysed cells. After RNA extraction, the RNA concentration was quantified with NanoDrop 2000 (Thermo Scientific), and sample purity was evaluated at A260/A280 (protein purity; ≈2.0+) and A260/A230 (salt purity; 2.0–2.2). RevertAid H Minus First Strand cDNA Synthesis Kit (Thermo Scientific, USA) was used for reverse transcription; 500 ng of RNA was mixed with 1 µg of oligo^(dT18)^ for each reaction (5 min at 65 °C). The reaction mixture (4 µL 5× reaction buffer, 1 µL ribolock ribonuclease inhibitor, 2 µL dNTPmix (10 × 10^−3^ m), and 1 µL RevertAid H Minus M‐MuL VRT) was added to the samples and run in Applied Biosystems Veriti 96‐Well Fast Thermal Cycler (60 min at 42 °C followed by 10 min at 70 °C). qRT‐PCR was further performed with the cDNA, SYBR Green (Life Technologies) and primers designed for the specific human markers in cycling: 95 °C, 3 s; 60 °C, 20 s. Glyceraldehyde 3‐phosphate dehydrogenase (GAPDH) was used as house‐keeping gene and relative differences in expression were calculated using the 2^−ΔΔ^ *^Ct^* method.^88^

*RNA Sequencing*: RNA isolation from samples (*n* = 3–4) was performed as described above. The Genomics Core Leuven performed the sequencing and the RNA‐seq expression analysis as follows. Library preparation was performed with the Illumina TruSeq Stranded mRNA Sample Preparation Kit, according to the manufacturer's protocol. Denaturation of RNA was performed at 65 °C in a thermocycler and cooled down to 4 °C. Samples were indexed to allow for multiplexing. Sequencing libraries were quantified using the Qubit fluorometer (Thermo Fisher Scientific, MA, USA). Library quality and size range were assessed using the Bioanalyzer (Agilent Technologies) with the DNA 1000 kit (Agilent Technologies, CA, USA) according to the manufacturer's recommendations. Each library was diluted to a final concentration of 2 × 10^−9^ m and sequenced on Illumina HiSeq4000 according to the manufacturer's recommendations generating 50 bp single‐end reads. A minimum of 14M reads per sample were produced. Quality control of raw reads was performed with FastQC v0.11.5. Adapters were filtered with ea‐utils v1.2.2.18. Splice‐aware alignment was performed with TopHat v2.0.13 against the human hg19. The number of allowed mismatches was 2. Reads that mapped to more than one site to the reference genome were discarded. The minimal score of alignment quality to be included in count analysis was 10. Resulting sequence alignment map (SAM) and binary alignment map (BAM) alignment files were handled with Samtools v0.1.19.24. Quantification of reads per gene was performed with HT‐Seq count v0.5.3p3. Count‐based differential expression analysis was done with R‐based (The *R* Foundation for Statistical Computing, Vienna, Austria) Bioconductor package DESeq. Reported *p*‐values were adjusted for multiple testing with the Benjamini–Hochberg procedure, which controls false discovery rate (FDR). A list of differentially expressed genes was selected at an FDR of 0.05.

*Formation of Microtissue Constructs*: Macrowells with a diameter and a depth of 2 mm (ectopic implantation) and a length of 5 mm, a width of 3 mm, and a depth of 2 mm (orthotopic implantation) were created with 3% w/v agarose (Invitrogen, Belgium) and sterilized under UV. Microtissues were recuperated from their microwells by gently pipetting up and down several times. The microtissue suspension was concentrated with centrifugation to a volume corresponding to the macrowells. Next, the microtissues were added into the macrowells (≈3000 for ectopic and ≈6000 for large bone defect implantation) and incubated for 1 h to sediment, where after CM was added and constructs were incubated for additional 23 h to fuse into constructs.

*In Vivo Implantation of Microtissue Constructs*: Subcutaneous implantation was used to validate the construct's autonomy to form cartilage and bone tissue. Bone and cartilage do not naturally form in this location and chondro‐ and osteo‐inductive signals must therefore arise from the construct itself. After 24 h fusion, the microtissue constructs were implanted subcutaneously in immune compromised mice (*Rj*:NMRI^nu/nu^). Explants were taken out 4 and 8 weeks after in vivo implantation and fixed in 4% PFA for subsequent nano‐CT and histological analysis. A large bone defect mouse model, described elsewhere,^58^ was used to assess the impact of the environment and mechanical loading on the bone forming potential of the day 21 microtissue constructs. Briefly, a custom‐made Ilizarov fixator was fixed to the tibia using 27 G steel needles. The tibia was exposed, and a 4 mm mid‐diaphyseal segment was removed with a diamond saw. Custom‐made constructs (≈6000 callus organoids per construct, *n* = 4) were placed into the defect, and the skin was sutured to close the wound. An empty defect was used as control (*n* = 4). Defects were monitored with in vivo micro‐CT (SkyScan 1076, Bruker micro‐CT, BE) 1, 2, 4, 6, and 8 weeks after surgery (voxel size of 9 µm). Animals were sacrificed after 8 weeks; the tibia was fixed in 4% PFA and analyzed with ex vivo nano‐CT and processed for histology. All procedures on animal experiments were approved by the local ethical committee for Animal Research, KU Leuven. The animals were housed according to the regulations of the Animalium Leuven (KU Leuven).

*Quantification of Mineralized Tissue from In Vivo Micro‐CT and Ex Vivo Nano‐CT*: Ex vivo nano‐CT (Pheonix Nanotom M, GE Measurement, and Control Solutions) was used for 3D quantification of mineralized tissue in each explant. Explants were scanned with a diamond target, mode 0, 500 ms exposure time, 1 frame average, 0 image skip, 2400 images, and a 0.2 mm aluminum filter. Subcutaneous explants were scanned at a voltage of 60 kV and a current of 140 µA resulting in a voxel size of 2 µm. Large bone defect explants and native tibia were scanned at a voltage of 60 kV and a current of 390 µA resulting in a voxel size of 5.6 µm. CTAn (Bruker micro‐CT, BE) was used for all image processing and quantification of mineralized tissue based on automatic Otsu segmentation, 3D space closing, and despeckle algorithm. Percentage of mineralized tissue was calculated with respect to the total explant volume. CTvox (Bruker micro‐CT, BE) was used to create 3D visualization.

*Histochemistry and Immuno‐Histochemistry*: Retrieved subcutaneous explants were fixed in 4% PFA overnight and decalcified in ethylenediaminetetraacetic acid (EDTA)/PBS (pH 7.5) for 10 days at 4 °C followed by paraffin embedding. Tibias were fixed in 2% PFA overnight and decalcified in EDTA/PBS (pH 7.5) for 3 weeks then dehydrated and embedded in paraffin. Ectopic samples were sectioned at 5 µm and tibias at 6 µm. Histology was performed according to previously reported methods of H&E, Alcian Blue, Masson's Trichrome, and Safranin O staining.^10^ Immuno‐histochemistry was performed on PFA‐fixed microtissues (Osterix), paraffin‐embedded PFA‐fixed microtissues (Indian Hedgehog), and paraffin‐embedded EDTA‐decalcified explants (human osteocalcin, CD31). Epitope retrieval was performed with Uni‐Trieve (INNOVEX Bioscience, USA) for 30 min at 70 °C. Quenching of endogenous peroxidase activity was performed with 3% H_2_O_2_ for 10 min. Next, sections were blocked in serum for 30 min and incubated overnight at 4 °C with the primary antibodies human osterix (R&D Systems, MAB7547: dilution 1:300), human osteocalcin^29^ (a gift from E. Van Herck, Legendo, KU Leuven, BE; dilution 1:5000), rabbit polyclonal anti‐Ihh antibody–N‐terminal (Abcam, ab80191; dilution 1:50), rabbit anticollagen type II (Merck Millipore, AB761; dilution 1:50), or purified rat antimouse CD31 (BD Biosciences, USA, 550 274; dilution 1:50). Next, slides were blocked and incubated with the secondary antibodies Alexa 488 antimouse (Thermo Fisher Scientific, A11001; dilution 1:500), horseradish peroxidase (HRP) conjugated goat anti‐guineaPig or—rabbit (Jackson ImmunoResearch, UK; dilution 1:500) for 30 min and peroxidase activity was determined using 3,3′‐diaminobenzidine (DAB) (K3468, Dako, USA). For detection of CD31, the secondary antibody Biotin conjugated Goat‐anti‐Rat Ig (BD Biosceinces, USA, 559 286) and a tyramide signal amplification (TSA) Biotin detection system (PerkinElmer, USA) were used. Stained histology sections were visualized with a Leica M165 FC microscope (Microsystems, Belgium) or an inverted laser scanning fluorescence confocal microscope ZEISS LSM 510 META (Cell imaging core facility of KU Leuven). Histomorphometry was performed in ImageJ software using ROI manager^87^ on three to four nonconsecutive sections per sample, and mean values from these sections were used as data point for one sample.

*Transcriptomics Analysis*: An unsupervised analysis of the RNA‐seq data and subsequently gene visualization was performed. For this, a [gene × experimental condition] matrix was obtained from the bulk RNA‐seq data. First genes were ranked based on variance, and then the gene expression profile of 400 most variable genes across four time points was selected for downstream analysis. Gene expression values were mean and log2‐normalized. Then, *k*‐means clustering was used to computationally cluster these genes based on their expression profiles.

In order to select the number of clusters, the elbow method was applied and determined that *k* = 5 was the optimal parameter for achieving the most robust partition. Clustering results were visualized in order to provide insight into the patterns of correlation between samples and expression levels. A profile plot, also known as parallel coordinate plot was plotted using ggplot2—a package for data visualization within the *R*‐statistical computing environment (http://www.r-project.org/) in order to visualize the expression levels of a total of 400 gene transcripts across all four time points including *k*‐means cluster information. Subsequently, Gene Ontology enrichment of Biological Processes (2017b) for each cluster was performed with Enrichr.^38^, ^39^

*Statistical Analysis*: All experiments were performed with at least three replicates per condition. Data were represented as mean ± standard error of the mean (SEM) or box‐plot with 10–90 percentiles, if otherwise not stated. Data were compared with one‐way or two‐way ANOVA and Tukey's Multiple Comparison test or Student's *t* test. Results were considered statistically different for *p‐*values lower than 0.05 (*p < 0.05, **p < 0.01, ***p < 0.001). Statistical analysis was performed with GraphPad Prism 8 (GraphPad Software, Inc., USA) unless otherwise stated.

## Conflict of Interest

The authors declare no conflict of interest.

## Supporting information

Supporting Information

Supplemental Movie 1

Supplemental Movie 2

Supporting Information

Supporting Information

Supporting Information

## References

[1] S. Dimmeler , S. Ding , T. A. Rando , A. Trounson , Nat. Med. 2014, 20, 814. 25100527 10.1038/nm.3627

[2] Y. Y. Lipsitz , N. E. Timmins , P. W. Zandstra , Nat. Biotechnol. 2016, 34, 393. 27054995 10.1038/nbt.3525

[3] D. C. Kirouac , P. W. Zandstra , Cell Stem Cell 2008, 3, 369. 18940729 10.1016/j.stem.2008.09.001

[4] P. Lenas , Regen. Med. 2018, 13, 7. 29369015 10.2217/rme-2017-0126

[5] P. Lenas , M. Moos , F. P. Luyten , Tissue Eng., Part B 2009, 15, 381. 10.1089/ten.TEB.2008.0575 19505199

[6] P. Lenas , F. P. Luyten , Ind. Eng. Chem. Res. 2011, 50, 482.

[7] R. S. Marcucio , L. Qin , E. Alsberg , J. D. Boerckel , J. Orthop. Res. 2017, 35, 2356. 28660712 10.1002/jor.23636

[8] T. Takebe , M. Enomura , E. Yoshizawa , M. Kimura , H. Koike , Y. Ueno , T. Matsuzaki , T. Yamazaki , T. Toyohara , K. Osafune , H. Nakauchi , H. Y. Yoshikawa , H. Taniguchi , Cell Stem Cell 2015, 16, 556. 25891906 10.1016/j.stem.2015.03.004

[9] N. C. Rivron , J. Frias‐Aldeguer , E. J. Vrij , J. C. Boisset , J. Korving , J. Vivié , R. K. Truckenmüller , A. Van Oudenaarden , C. A. Van Blitterswijk , N. Geijsen , Nature 2018, 557, 106. 29720634 10.1038/s41586-018-0051-0

[10] W. A. Fernando , I. Papantoniou , L. F. Mendes , G. Nilsson Hall , K. Bosmans , W. L. Tam , L. M. Teixeira , M. Moos , L. Geris , F. P. Luyten , J. Tissue Eng. Regener. Med. 2017, 12, 794. 10.1002/term.2498 28603948

[11] M. Huch , H. Gehart , R. Van Boxtel , K. Hamer , F. Blokzijl , M. M. A. Verstegen , E. Ellis , M. Van Wenum , S. A. Fuchs , J. De Ligt , M. van de Wetering , N. Sasaki , S. J. Boers , H. Kemperman , J. de Jonge , J. N. Ijzermans , E. E. Nieuwenhuis , R. Hoekstra , S. Strom , R. R. Vries , L. J. van der Laan , E. Cuppen , H. Clevers , Cell 2015, 160, 299. 25533785 10.1016/j.cell.2014.11.050 PMC4313365

[12] T. a. Einhorn , L. C. Gerstenfeld , Nat. Rev. Rheumatol. 2014, 11, 45. 25266456 10.1038/nrrheum.2014.164 PMC4464690

[13] H. Kronenberg , Nature 2003, 423, 332. 12748651 10.1038/nature01657

[14] C. Colnot , J. Bone Miner. Res. 2009, 24, 274. 18847330 10.1359/jbmr.081003 PMC3276357

[15] O. Duchamp de Lageneste , A. Julien , R. Abou‐Khalil , G. Frangi , C. Carvalho , N. Cagnard , C. Cordier , S. J. Conway , C. Colnot , Nat. Commun. 2018, 9, 773. 29472541 10.1038/s41467-018-03124-z PMC5823889

[16] J. M. Jukes , S. K. Both , A. Leusink , L. M. T. Sterk , C. A. van Blitterswijk , J. de Boer , Proc. Natl. Acad. Sci. USA 2008, 105, 6840. 18467492 10.1073/pnas.0711662105 PMC2374550

[17] C. Scotti , B. Tonnarelli , A. Papadimitropoulos , A. Scherberich , S. Schaeren , A. Schauerte , J. Lopez‐Rios , R. Zeller , A. Barbero , I. Martin , Proc. Natl. Acad. Sci. USA 2010, 107, 7251. 20406908 10.1073/pnas.1000302107 PMC2867676

[18] C. Scotti , E. Piccinini , H. Takizawa , A. Todorov , P. Bourgine , A. Papadimitropoulos , A. Barbero , M. G. Manz , I. Martin , Proc. Natl. Acad. Sci. USA 2013, 110, 3997. 23401508 10.1073/pnas.1220108110 PMC3593845

[19] N. Harada , Y. Watanabe , K. Sato , S. Abe , K. Yamanaka , Y. Sakai , T. Kaneko , T. Matsushita , Biomaterials 2014, 35, 7800. 24952976 10.1016/j.biomaterials.2014.05.052

[20] A. M. McDermott , S. Herberg , D. E. Mason , J. M. Collins , H. B. Pearson , J. H. Dawahare , R. Tang , A. N. Patwa , M. W. Grinstaff , D. J. Kelly , E. Alsberg , J. D. Boerckel , Sci. Transl. Med. 2019, 11, eaav7756. 31167930 10.1126/scitranslmed.aav7756 PMC6959418

[21] A. Atala , F. K. Kasper , A. G. Mikos , Sci. Transl. Med. 2012, 4, 160rv12. 10.1126/scitranslmed.3004890 23152327

[22] M. W. Laschke , M. D. Menger , Trends Biotechnol. 2017, 35, 133. 27634310 10.1016/j.tibtech.2016.08.004

[23] K. Jakab , A. Neagu , V. Mironov , R. R. Markwald , G. Forgacs , Proc. Natl. Acad. Sci. USA 2004, 101, 2864. 14981244 10.1073/pnas.0400164101 PMC365711

[24] V. Mironov , R. P. Visconti , V. Kasyanov , G. Forgacs , C. J. Drake , R. R. Markwald , Biomaterials 2009, 30, 2164. 19176247 10.1016/j.biomaterials.2008.12.084 PMC3773699

[25] K. Futrega , J. S. Palmer , M. Kinney , W. B. Lott , M. D. Ungrin , P. W. Zandstra , M. R. Doran , Biomaterials 2015, 62, 1. 26010218 10.1016/j.biomaterials.2015.05.013

[26] A. Leferink , D. Schipper , E. Arts , E. Vrij , N. Rivron , M. Karperien , K. Mittmann , C. van Blitterswijk , L. Moroni , R. Truckenmüller , Adv. Mater. 2014, 26, 2592. 24395427 10.1002/adma.201304539

[27] E. Fennema , N. Rivron , J. Rouwkema , C. van Blitterswijk , J. De Boer , Trends Biotechnol. 2013, 31, 108. 23336996 10.1016/j.tibtech.2012.12.003

[28] A. D. Dikina , D. S. Alt , S. Herberg , A. McMillan , H. A. Strobel , Z. Zheng , M. Cao , B. P. Lai , O. Jeon , V. I. Petsinger , C. U. Cotton , M. W. Rolle , E. Alsberg , Adv. Sci. 2018, 5, 1700402. 10.1002/advs.201700402 PMC5978945 29876200

[29] C. De Bari , F. Dell'Accio , J. Vanlauwe , J. Eyckmans , I. M. Khan , C. W. Archer , E. a. Jones , D. McGonagle , T. a. Mitsiadis , C. Pitzalis , F. P. Luyten , Arthritis Rheum. 2006, 54, 1209. 16575900 10.1002/art.21753

[30] K. M. Pineault , J. Y. Song , K. M. Kozloff , D. Lucas , D. M. Wellik , Nat. Commun. 2019, 10, 3168. 31320650 10.1038/s41467-019-11100-4 PMC6639390

[31] E. J. Mackie , Y. a. Ahmed , L. Tatarczuch , K.‐S. Chen , M. Mirams , Int. J. Biochem. Cell Biol. 2008, 40, 46. 17659995 10.1016/j.biocel.2007.06.009

[32] D. Kobayashi , T. Oike , A. Shibata , A. Niimi , Y. Kubota , M. Sakai , N. Amornwhichet , Y. Yoshimoto , Y. Hagiwara , Y. Kimura , Y. Hirota , H. Sato , M. Isono , Y. Yoshida , T. Kohno , T. Ohno , T. Nakano , Sci. Rep. 2017, 7, 40588. 28091564 10.1038/srep40588 PMC5238371

[33] L. Yang , K. Y. Tsang , H. C. Tang , D. Chan , K. S. E. Cheah , World Rev. Nutr. Diet. 2014, 111, 13.25418383

[34] X. Zhou , Z. Zhang , J. Q. Feng , V. M. Dusevich , K. Sinha , H. Zhang , B. G. Darnay , B. de Crombrugghe , Proc. Natl. Acad. Sci. USA 2010, 107, 12919. 20615976 10.1073/pnas.0912855107 PMC2919908

[35] C. S. Bahney , D. P. Hu , T. Miclau , R. S. Marcucio , Front. Endocrinol. 2015, 6, 4. 10.3389/fendo.2015.00004 PMC4318416 25699016

[36] J. Park , M. Gebhardt , S. Golovchenko , F. Perez‐Branguli , T. Hattori , C. Hartmann , X. Zhou , B. deCrombrugghe , M. Stock , H. Schneider , K. von der Mark , Biol. Open 2015, 4, 608. 25882555 10.1242/bio.201411031 PMC4434812

[37] E. Holm , J. E. Aubin , G. K. Hunter , F. Beier , H. A. Goldberg , Bone 2015, 71, 145. 25464126 10.1016/j.bone.2014.10.007

[38] E. Y. Chen , C. M. Tan , Y. Kou , Q. Duan , Z. Wang , G. V. Meirelles , N. R. Clark , A. Ma'ayan , BMC Bioinformatics 2013, 14, 128. 23586463 10.1186/1471-2105-14-128 PMC3637064

[39] M. V. Kuleshov , M. R. Jones , A. D. Rouillard , N. F. Fernandez , Q. Duan , Z. Wang , S. Koplev , S. L. Jenkins , K. M. Jagodnik , A. Lachmann , M. G. McDermott , C. D. Monteiro , G. W. Gundersen , A. Ma'ayan , Nucleic Acids Res. 2016, 44, W90. 27141961 10.1093/nar/gkw377 PMC4987924

[40] M. Kutmon , A. Riutta , N. Nunes , K. Hanspers , E. L. Willighagen , A. Bohler , J. Mélius , A. Waagmeester , S. R. Sinha , R. Miller , S. L. Coort , E. Cirillo , B. Smeets , C. T. Evelo , A. R. Pico , Nucleic Acids Res. 2016, 44, D488. 26481357 10.1093/nar/gkv1024 PMC4702772

[41] W. E. Samsa , X. Zhou , G. Zhou , Semin. Cell Dev. Biol. 2017, 62, 3. 27418125 10.1016/j.semcdb.2016.07.008 PMC5226926

[42] T. J. Mead , K. E. Yutzey , Proc. Natl. Acad. Sci. USA 2009, 106, 14420. 19590010 10.1073/pnas.0902306106 PMC2732875

[43] K. Yeung Tsang , S. Wa Tsang , D. Chan , K. S. E. Cheah , Birth Defects Res., Part C 2014, 102, 52. 10.1002/bdrc.21060 24677723

[44] J. P. Iannotti , C. T. Brighton , J. Orthop. Res. 1989, 7, 511. 2738769 10.1002/jor.1100070408

[45] A. R. Guntur , C. J. Rosen , BoneKEy Rep. 2013, 2, 437. 24422135 10.1038/bonekey.2013.171 PMC3818534

[46] M. Barna , P. P. Pandolfi , L. Niswander , Nature 2005, 436, 277. 16015334 10.1038/nature03801

[47] M. B. Goldring , K. Tsuchimochi , K. Ijiri , J. Cell. Biochem. 2006, 97, 33. 16215986 10.1002/jcb.20652

[48] Z. Tan , B. Niu , K. Y. Tsang , I. G. Melhado , S. Ohba , X. He , Y. Huang , C. Wang , A. P. McMahon , R. Jauch , D. Chan , M. Q. Zhang , K. S. E. Cheah , PLoS Genet. 2018, 14, 1007346. 10.1371/journal.pgen.1007346 PMC5919691 29659575

[49] H. M. Kronenberg , Ann. N. Y. Acad. Sci. 2006, 1068, 1. 16831900 10.1196/annals.1346.002

[50] P. Smits , P. Dy , S. Mitra , V. Lefebvre , J. Cell Biol. 2004, 164, 747. 14993235 10.1083/jcb.200312045 PMC2172159

[51] J. Li , H. Luo , R. Wang , J. Lang , S. Zhu , Z. Zhang , J. Fang , K. Qu , Y. Lin , H. Long , Y. Yao , G. Tian , Q. Wu , Cell Rep. 2016, 15, 1467. 27160914 10.1016/j.celrep.2016.04.043

[52] A. Ionescu , E. Kozhemyakina , C. Nicolae , K. H. Kaestner , B. R. Olsen , A. B. Lassar , Dev. Cell 2012, 22, 927. 22595668 10.1016/j.devcel.2012.03.011 PMC3356573

[53] L. Ye , Y. Mishina , D. Chen , H. Huang , S. L. Dallas , M. R. Dallas , P. Sivakumar , T. Kunieda , T. W. Tsutsui , A. Boskey , L. F. Bonewald , J. Q. Feng , J. Biol. Chem. 2005, 280, 6197. 15590631 10.1074/jbc.M412911200 PMC2647591

[54] M. K. Song , Z. H. Lee , H. H. Kim , Exp. Mol. Med. 2015, 47, e199. 26642432 10.1038/emm.2015.94 PMC4686697

[55] Y. Nakamichi , C. Shukunami , T. Yamada , K.‐i. Aihara , H. Kawano , T. Sato , Y. Nishizaki , Y. Yamamoto , M. Shindo , K. Yoshimura , T. Nakamura , N. Takahashi , H. Kawaguchi , Y. Hiraki , S. Kato , Mol. Cell. Biol. 2003, 23, 636. 12509461 10.1128/MCB.23.2.636-644.2003 PMC151528

[56] S. Miura , J. Kondo , A. Takimoto , H. Sano‐Takai , L. Guo , C. Shukunami , H. Tanaka , Y. Hiraki , PLoS One 2014, 9, 3. 10.1371/journal.pone.0094239 PMC3977995 24710035

[57] H. H. Lee , R. R. Behringer , PLoS One 2007, 2, 450. 10.1371/journal.pone.0000450 PMC1865390 17505543

[58] N. Van Gastel , S. Stegen , I. Stockmans , K. Moermans , J. Schrooten , D. Graf , F. P. Luyten , G. Carmeliet , Stem Cells 2014, 32, 2407. 24989687 10.1002/stem.1783

[59] S. Debnath , A. R. Yallowitz , J. McCormick , S. Lalani , T. Zhang , R. Xu , N. Li , Y. Liu , Y. S. Yang , M. Eiseman , J.‐H. Shim , M. Hameed , J. H. Healey , M. P. Bostrom , D. A. Landau , M. B. Greenblatt , Nature 2018, 562, 133. 30250253 10.1038/s41586-018-0554-8 PMC6193396

[60] C. S. Bahney , D. P. Hu , A. J. Taylor , F. Ferro , H. M. Britz , B. Hallgrimsson , B. Johnstone , T. Miclau , R. S. Marcucio , J. Bone Miner. Res. 2014, 29, 1269. 24259230 10.1002/jbmr.2148 PMC4802866

[61] J. van der Stok , M. K. E. Koolen , H. Jahr , N. Kops , J. H. Waarsing , H. Weinans , O. P. van der Jagt , Eur. Cells Mater. 2014, 27, 137. 10.22203/ecm.v027a11 24554271

[62] C. Epple , A. Haumer , T. Ismail , A. Lunger , A. Scherberich , D. J. Schaefer , I. Martin , Biomaterials 2018, 192, 118. 30448696 10.1016/j.biomaterials.2018.11.008

[63] P. Li , J. S. Markson , S. Wang , S. Chen , V. Vachharajani , M. B. Elowitz , Science 2018, 360, 543. 29622726 10.1126/science.aao0645 PMC6516753

[64] J. Groll , J. A. Burdick , D.‐W. Cho , B. Derby , M. Gelinsky , S. C. Heilshorn , T. Jüngst , J. Malda , V. A. Mironov , K. Nakayama , A. Ovsianikov , W. Sun , S. Takeuchi , J. J. Yoo , T. B. F. Woodfield , Biofabrication 2018, 11, 013001. 30468151 10.1088/1758-5090/aaec52

[65] A. Page‐McCaw , A. J. Ewald , Z. Werb , Nat. Rev. Mol. Cell Biol. 2007, 8, 221. 17318226 10.1038/nrm2125 PMC2760082

[66] C. K. F. Chan , C.‐C. Chen , C. A. Luppen , J.‐B. Kim , A. T. DeBoer , K. Wei , J. A. Helms , C. J. Kuo , D. L. Kraft , I. L. Weissman , Nature 2009, 457, 490. 19078959 10.1038/nature07547 PMC2648141

[67] A. Ovsianikov , A. Khademhosseini , V. Mironov , Trends Biotechnol. 2018, 36, 348. 29475621 10.1016/j.tibtech.2018.01.005

[68] A. C. Daly , P. Pitacco , J. Nulty , G. M. Cunniffe , D. J. Kelly , Biomaterials 2018, 162, 34. 29432987 10.1016/j.biomaterials.2018.01.057

[69] L. Moroni , J. A. Burdick , C. Highley , S. J. Lee , Y. Morimoto , S. Takeuchi , J. J. Yoo , Nat. Rev. Mater. 2018, 3, 21. 31223488 10.1038/s41578-018-0006-y PMC6586020

[70] A. Petersen , A. Princ , G. Korus , A. Ellinghaus , H. Leemhuis , A. Herrera , A. Klaumünzer , S. Schreivogel , A. Woloszyk , K. Schmidt‐Bleek , S. Geissler , I. Heschel , G. N. Duda , Nat. Commun. 2018, 9, 4430. 30361486 10.1038/s41467-018-06504-7 PMC6202397

[71] F. Qu , J. L. Holloway , J. L. Esterhai , J. A. Burdick , R. L. Mauck , Nat. Commun. 2017, 8, 1780. 29176654 10.1038/s41467-017-01955-w PMC5701126

[72] A. W. James , G. LaChaud , J. Shen , G. Asatrian , V. Nguyen , X. Zhang , K. Ting , C. Soo , Tissue Eng., Part B 2016, 22, 284. 10.1089/ten.teb.2015.0357 PMC4964756 26857241

[73] S. Herberg , A. M. McDermott , P. N. Dang , D. S. Alt , R. Tang , J. H. Dawahare , D. Varghai , J.‐Y. Shin , A. McMillan , A. D. Dikina , F. He , Y. B. Lee , Y. Cheng , K. Umemori , P. C. Wong , H. Park , J. D. Boerckel , E. Alsberg , Sci. Adv. 2019, 5, eaax2476. 31489377 10.1126/sciadv.aax2476 PMC6713501

[74] Z. A. Cheng , A. Alba‐Perez , C. Gonzalez‐Garcia , H. Donnelly , V. Llopis‐Hernandez , V. Jayawarna , P. Childs , D. W. Shields , M. Cantini , L. Ruiz‐Cantu , A. Reid , J. F. C. Windmill , E. S. Addison , S. Corr , W. G. Marshall , M. J. Dalby , M. Salmeron‐Sanchez , Adv. Sci. 2019, 6, 1800361. 10.1002/advs.201800361 PMC6343071 30693176

[75] A. Lang , M. Kirchner , J. Stefanowski , M. Durst , M. C. Weber , M. Pfeiffenberger , A. Damerau , A. E. Hauser , P. Hoff , G. N. Duda , F. Buttgereit , K. Schmidt‐Bleek , T. Gaber , Acta Biomater. 2019, 86, 171. 30616076 10.1016/j.actbio.2018.12.043

[76] H. Fonoudi , H. Ansari , S. Abbasalizadeh , M. R. Larijani , S. Kiani , S. Hashemizadeh , A. S. Zarchi , A. Bosman , G. M. Blue , S. Pahlavan , M. Perry , Y. Orr , Y. Mayorchak , J. Vandenberg , M. Talkhabi , D. S. Winlaw , R. P. Harvey , N. Aghdami , H. Baharvand , Stem Cells Transl. Med. 2015, 4, 1482. 26511653 10.5966/sctm.2014-0275 PMC4675501

[77] S. Sart , R. F. X. Tomasi , G. Amselem , C. N. Baroud , Nat. Commun. 2017, 8, 469. 28883466 10.1038/s41467-017-00475-x PMC5589863

[78] S. V. Murphy , A. Atala , Nat. Biotechnol. 2014, 32, 773. 25093879 10.1038/nbt.2958

[79] N. I. Moldovan , N. Hibino , K. Nakayama , Tissue Eng., Part B 2017, 23, 237. 10.1089/ten.TEB.2016.0322 27917703

[80] N. V. Mekhileri , K. S. Lim , G. C. J. Brown , I. Mutreja , B. S. Schon , G. J. Hooper , T. B. F. Woodfield , Biofabrication 2018, 10, 024103. 29199637 10.1088/1758-5090/aa9ef1

[81] J. Rouwkema , A. Khademhosseini , Trends Biotechnol. 2016, 34, 733. 27032730 10.1016/j.tibtech.2016.03.002

[82] M. A. Skylar‐Scott , S. G. M. Uzel , L. L. Nam , J. H. Ahrens , R. L. Truby , S. Damaraju , J. A. Lewis , Sci. Adv. 2019, 5, eaaw2459. 31523707 10.1126/sciadv.aaw2459 PMC6731072

[83] C. K. F. Chan , G. S. Gulati , R. Sinha , J. V. Tompkins , M. Lopez , A. C. Carter , R. C. Ransom , A. Reinisch , T. Wearda , M. Murphy , R. E. Brewer , L. S. Koepke , O. Marecic , A. Manjunath , E. Y. Seo , T. Leavitt , W.‐J. Lu , A. Nguyen , S. D. Conley , A. Salhotra , T. H. Ambrosi , M. R. Borrelli , T. Siebel , K. Chan , K. Schallmoser , J. Seita , D. Sahoo , H. Goodnough , J. Bishop , M. Gardner , R. Majeti , D. C. Wan , S. Goodman , I. L. Weissman , H. Y. Chang , M. T. Longaker , Cell 2018, 175, 43. 30241615 10.1016/j.cell.2018.07.029 PMC6400492

[84] J. Eyckmans , S. J. Roberts , J. Schrooten , F. P. Luyten , J. Cell. Mol. Med. 2010, 14, 1845. 19538476 10.1111/j.1582-4934.2009.00807.x PMC3829044

[85] J. Leijten , L. S. Moreira Teixeira , J. Bolander , W. Ji , B. Vanspauwen , J. Lammertyn , J. Schrooten , F. P. Luyten , Sci. Rep. 2016, 6, 36011. 27808102 10.1038/srep36011 PMC5093556

[86] L. F. Mendes , W. L. Tam , Y. C. Chai , L. Geris , F. P. Luyten , S. J. Roberts , Tissue Eng., Part C 2016, 22, 473. 10.1089/ten.TEC.2015.0436 27018617

[87] C. A. Schneider , W. S. Rasband , K. W. Eliceiri , Nat. Methods 2012, 9, 671. 22930834 10.1038/nmeth.2089 PMC5554542

[88] K. J. Livak , T. D. Schmittgen , Methods 2001, 25, 402. 11846609 10.1006/meth.2001.1262

