# Supplementary material for: Developmentally Engineered Callus Organoid Bioassemblies Exhibit Predictive In Vivo Long Bone Healing
Source: Adv Sci (Weinh). 2019 Dec 10;7(2):1902295. doi: 10.1002/advs.201902295 (PMC6974953; doi:10.1002/advs.201902295)
Supplement: Supplementary file 1 — Supporting Information [file ADVS-7-1902295-s001.pdf]

Copyright WILEY-VCH Verlag GmbH & Co. KGaA, 69469 Weinheim, Germany, 2019.

# Supporting Information

for *Adv. Sci.,* DOI: 10.1002/advs.201902295

## Developmentally Engineered Callus Organoid Bioassemblies Exhibit Predictive In Vivo Long Bone Healing

*Gabriella Nilsson Hall, Luís Freitas Mendes, Charikleia Gklava, Liesbet Geris,*
*Frank P. Luyten,* and Ioannis Papantoniou**

Copyright WILEY-VCH Verlag GmbH & Co. KGaA, 69469 Weinheim, Germany, 2019.

# Supporting Information

**Developmentally Engineered Callus Organoid Bioassemblies Exhibit Predictive In Vivo Long Bone Healing**

*Gabriella Nilsson Hall, Luís Filipe. Mendes, Charikleia Gklava, Liesbet Geris, Frank P. Luyten* [†, *], *Ioannis Papantoniou* [†, *]

[†] These authors share senior authorship

Supporting Figures

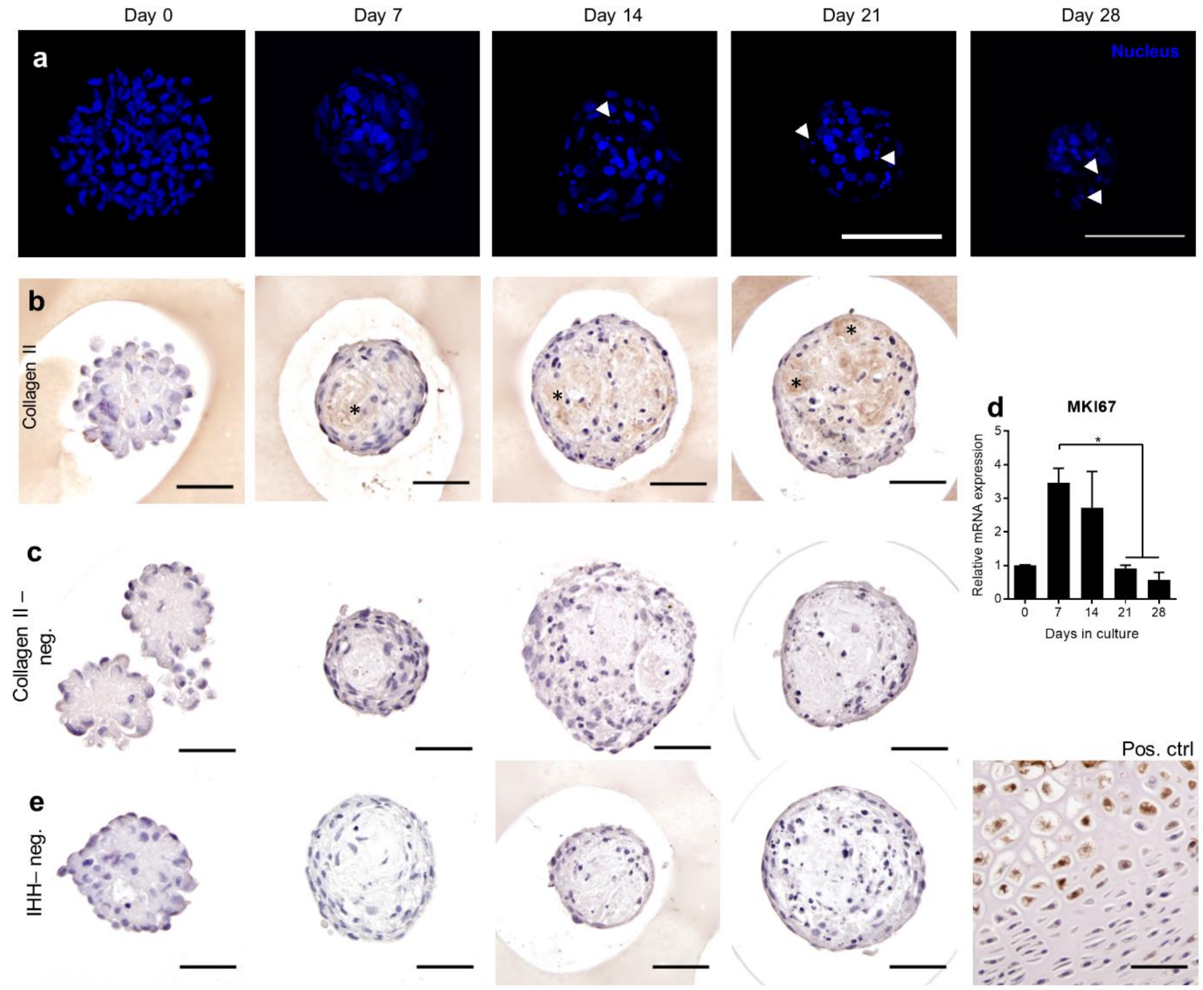


**Figure S1**. Characterization of cartilage intermediate microtissues. a) 3D renderings of confocal images of micro-spheroids stained with DAPI (nucleus) over time (white arrows represent apoptotic-like nuclei). b) Representative sections of collagen II immunostaining with c) negative control (excluding primary antibody). d) Quantification of MKI67 mRNA transcript ($n$ = 3, mean value ± SEM). *$p < 0.05$; ANOVA and Tukey's multiple comparisons test. e) Negative control (excluding primary antibody, left panels D0, 7, 14 and 21) and positive control (E14.5 mouse limb, right panel) for IHH immunostaining. Scale bar a: 100 µm; b, d-e: 50 µm.

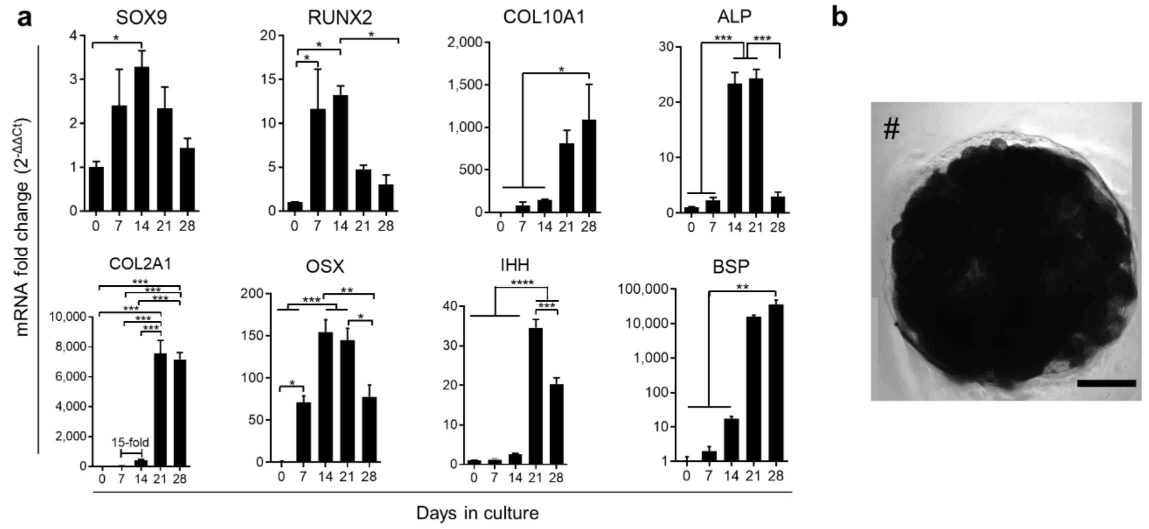


**Figure S2.** a) Gene expression analysis of spheroids overtime until D28. mRNA transcript quantification normalized to D0 (*n* = 3). b) Top-view of assembled modules within the in-house designed agarose mold (#: agarose). *p < 0.05; **p < 0.01; ***p < 0.001; ****p < 0.0001; ANOVA and Tukey's multiple comparisons test. Scale bar 500 µm.

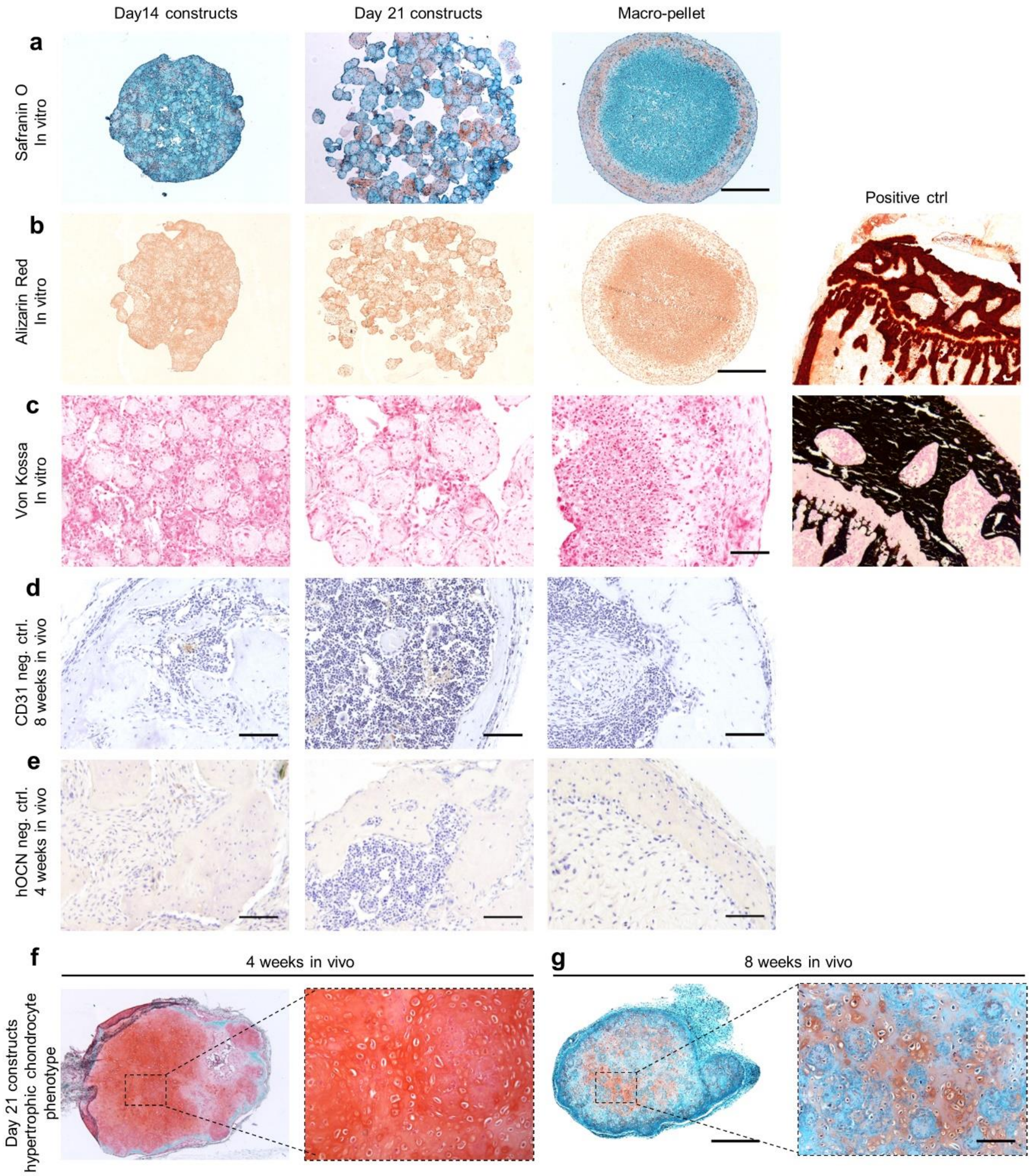


**Figure S3.** Assembly of callus organoids results in homogenous constructs able to form bone *in vivo*. a) Safranin O staining of constructs analyzed: Day 14, day 21 constructs and Macro-pellet. b) Alizarin Red staining and c) Von Kossa staining with positive control demonstrated lack of mineralization in fused constructs and Macro-pellet *in vitro*. d) Negative control (excluding primary antibody) of CD31 immunostaining after 8 weeks *in vivo* and e) hOCN immunostaining after 4 weeks *in vivo*. f-g) Safranin O staining of day 21 constructs after 4 and 8 weeks *in vivo*, representing a hypertrophic phenotype. Scale bars a-b: 500 µm, c-e: 100 µm, f-g: 500 µm (left) and 100 µm (right).

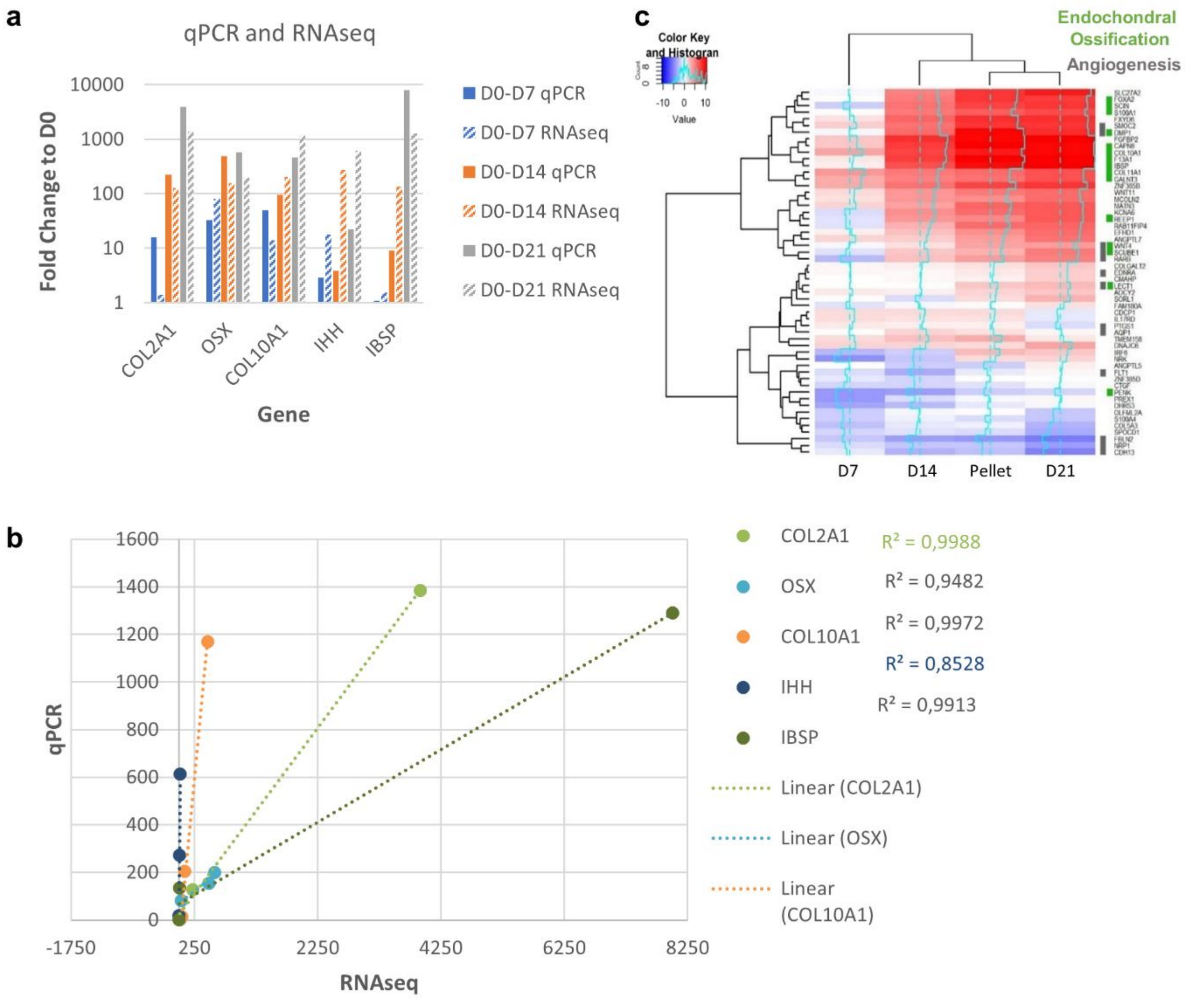


**Figure S4**. RNA sequencing analysis of callus organoid maturation. a) Comparison of key mRNA transcripts quantified with qPCR and RNA-seq demonstrating b) high linear fit for the two methods. c) Heat map of the 55 differentially expressed genes between D14 and D21 spheroids (green dots, Figure 5a) represented overtime and associated with endochondral ossification (green bar) and/or angiogenesis (grey bar).

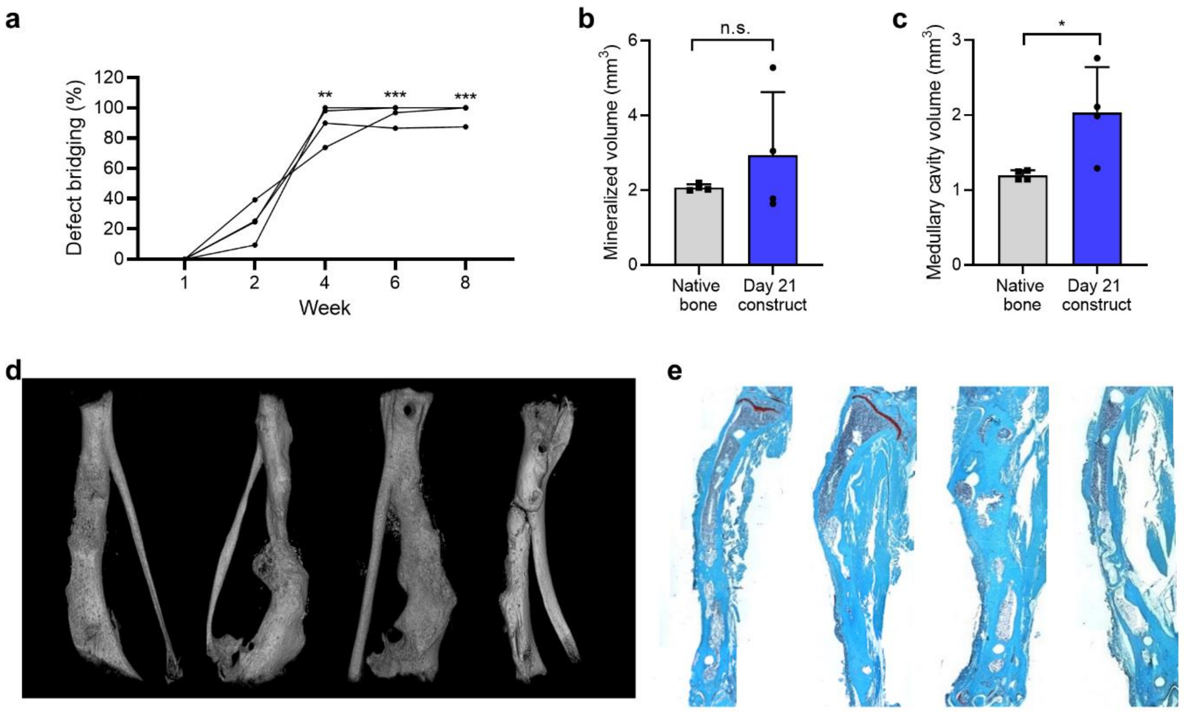


**Figure S5**. Fused callus organoids (Day 21 construct) heal critical-sized long bone defects in mice ($n$ = 4 animals). a) X-ray analysis of bone bridging over time defined by mineral bridging of the defect on two sides of the defect. ANOVA and Tukey's multiple comparisons test. **p < 0.01; ***p < 0.001 compared to Week 1. b) Comparison between native tibia and healed defect 8 weeks after construct implantation demonstrated by ex vivo nano-CT quantification of b) mineralized volume ($mm^3$) and c) medullary cavity volume ($mm^3$); unpaired t-test; *p < 0.05. d) 3D reconstruction of nano-CT scans and e) histological Safranin O staining for all four animals 8 weeks after construct implantation.

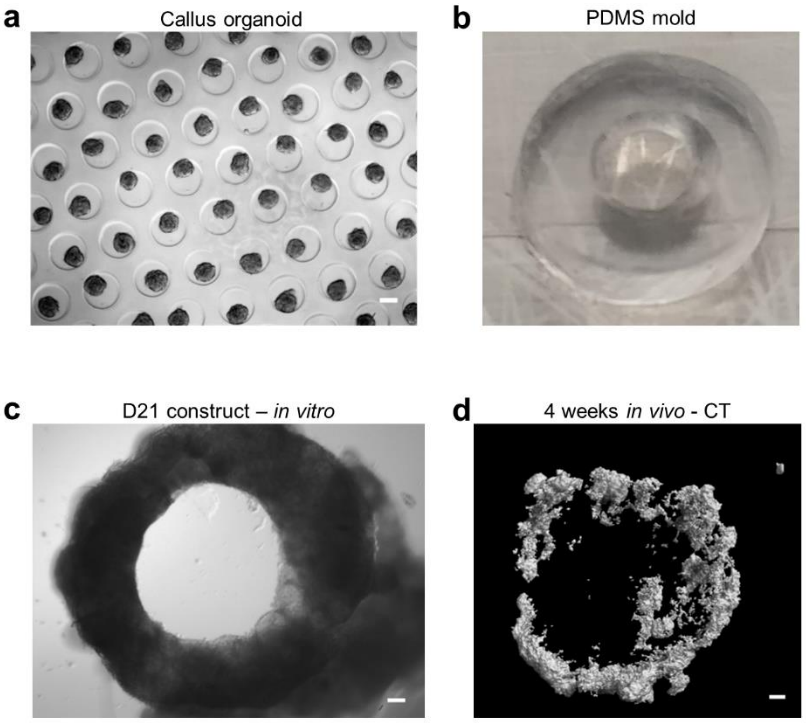


**Figure S6**. Formation of a ring-shaped mineralized tissue. a) callus organoids cultured in micro-wells for seeding in a ring-shaped agarose well made from b) an in-house designed PDMS mold. c) Mature callus organoids (D21) were assembled in the agarose well and fused for 24h resulting into a ring-shaped construct. d) A mineralized ring was formed after 4 weeks ectopic implantation. Scale bars a, c-d: 100 µm.

Supporting Tables

**Table S1.** Genes significantly up-regulated D14-21 and associated with endochondral ossification.

| Gene | Fold Change | Adj.p-value | Full name | Description | Localization |
|---|---|---|---|---|---|
| IBSP | 9,64 | 0,00017 | Bone Sialoprotein | Regulates both chondrocyte proliferation and apoptosis as well as transition from cartilage to bone during development of endochondral bone. [1] | Extracellular |
| LECT1 | 7,47 | 0,00015 | Chondromodulin (CNMD) | Expressed in the avascular zone of prehypertrophic cartilage and its expression decreases during chondrocyte hypertrophy and vascular invasion. The mature protein likely plays a role in endochondral bone development by permitting cartilaginous anlagen to be vascularized and replaced by bone. [2] | Extracellular |
| WNT4 | 6,65 | 0,03795 | Wnt Family Member 4 | Wnt4 accelerates chondrocyte differentiation. [3] | Plasma membrane, extracellular |
| RARB | 6,50 | 0,012 | Retinoic Acid Receptor Beta | Retinoid signaling crucial for chondrocyte maturation and endochondral ossification. [4] | Nucleus |
| FOXA2 | 6,41 | 0,00181 | Forkhead Box A2 | Detected in hypertrophic zone and mice engineered to lack expression of both FoxA2 and FoxA3 in their chondrocytes display defects in chondrocyte hypertrophy. [5] | |
| REEP1 | 6,35 | 0,00002 | Receptor Accessory Protein 1 | Increased expression in growth plate cartilage as compared to articular cartilage. [6] | endoplasmic reticulum |
| F13A1 | 6,23 | 0,00626 | Coagulation Factor XIII A Chain | Increased expression in the hypertrophic zone compared to the proliferative/resting zone of the growth plate. [7] | extracellular |
| COL10A1 | 5,81 | 0,00045 | Collagen Type X Alpha 1 Chain | ECM component secreted by hypertrophic chondrocytes. [8] | Extracellular, endoplasmic reticulum |
| DMP1 | 5,61 | 0,00002 | Dentin Matrix Acidic Phosphoprotein 1 | Critical for proper mineralization of bone. [9,10] | Extracellular, endoplasmic reticulum |
| SCIN | 5,31 | 0,00017 | Scinderin | Restricted to hypertrophic chondrocytes of the embryonic growth plate. Crucial for bone resorption. [11] | Extracellular |
| PENK | 4,89 | 0,00000 | Proenkephalin | Opioid. Expressed in the skeletal tissues, bone, and cartilage. Inhibit ALP expression in osteoblasts. [12] | Extracellular, endoplasmic reticulum |
| CAPN6 | 4,48 | 0,00004 | Calpain 6 | Calcium-dependent cysteine protease. Involved in proteoglycan degradation during bone development and fracture healing (rat). [13] | Cytoskeleton, cytosol |
| S100A1 | 4,10 | 0,00014 | S100 Calcium Binding Protein A1 | Transcriptional target of SOX trio and is present in late proliferative and prehypertrophic chondrocytes of mice growth plate. [14] | Extracellular, nucleus, endoplasmic reticulum |
| GALNT3 | 3,56 | 0,00262 | Polypeptide N-Acetylgalactosaminyltransferase 3 | Runx2 target gene in chondrocytes. Galnt3 is also likely to regulate chondrocyte maturation by modifying the glycosylation of Acan. [15] | Extracellular, golgi apparatus |
| COL11A1 | 2,91 | 0,01385 | Collagen Type XI Alpha 1 Chain | A fibrillar collagen gene, critically involved in spatial organization of growth plate chondrocytes. [16] | Extracellular, endoplasmic reticulum |

**Table S2**. Genes significantly up-regulated D14-21 and associated with angiogenesis.

| Gene | Fold Change | Adj.p-value | Full name | Description | Localization |
|---|---|---|---|---|---|
| IRF6 | 17,55 | 0,0065 | Interferon regulatory factor 6 | Transcription factor that plays a role in late endothelial progenitor cells. [17] | Nucleus, cytoplasm, extracellular |
| ANGPTL7 | 13,87 | 5,16E-16 | Angiopoietin Like 7 | Stimulate endothelial cell proliferation *in vitro* and vascularization *in vivo*. [18] | Extracellular |
| FLT1 (VEGFR-1) | 12,38 | 0,00046 | Vascular Permeability Factor Receptor | Receptor for vascular endothelial growth factor (VEGF), an essential mediator of angiogenesis. [19] | Plasma membrane, extracellular, cytoskeleton, endosome |
| SCUBE1 | 8,76 | 1,29E-06 | Signal Peptide, CUB Domain and EGF Like Domain Containing 1 | Cell surface glycoprotein associated to vascular biology. [20] | Plasma membrane, extracellular |
| LECT1 (CNMD) | 7,47 | 0,00015 | Chondromodulin | Expressed in the avascular zone of prehypertrophic cartilage. The mature protein likely plays a role in endochondral bone development by permitting cartilaginous anlagen to be vascularized and replaced by bone. [2] | Extracellular |
| WNT4 | 6,65 | 0,038 | Wnt Family Member 4 | Promote MSC-mediated angiogenesis. [21] | Plasma membrane, extracellular |
| RARB | 6,50 | 0,012 | Retinoic Acid Receptor Beta | | Nucleus |
| SMOC2 | 5,76 | 0,00032 | SPARC Related Modular Calcium Binding 2 | Angiogenic factor that potentiates angiogenic effects of growth factors. [22] | Extracellular |
| DMP1 | 5,61 | 0,00002 | Dentin Matrix Acidic Phosphoprotein 1 | Suggested as an inhibitor of VEGF-induced angiogenesis. [23] | Extracellular, endoplasmic reticulum |
| EDNRA | 3,12 | 0,0081 | Endothelin Receptor Type A | EDNRA is involved in the inhibition of angiogenesis in the retina. [24] | Plasma membrane |
| AQP1 | 0,36 | 0,045 | Aquaporin 1 | Targeted AQP1 gene disruption in mice reduces angiogenesis *in vivo*. [25] | Plasma membrane, extracellular, nucleus |
| FBLN2 | 0,34 | 0,041 | Fibulin 2 | Suppress angiogenesis in tumors *in vivo*. [26] | Extracellular |
| NRP1 | 0,27 | 0,017 | Neuropilin 1 | Stimulates angiogenesis in endothelial cells *in vitro*. [27] | Plasma membrane, extracellular |
| PTGS1 (COX-1) | 0,25 | 0,00047 | Prostaglandin-Endoperoxide Synthase 1 | Regulates angiogenesis in endothelial cells *in vitro*. [28] | Extracellular, nucleus, ER, golgi |
| CDH13 | 0,23 | 0,00046 | Cadherin 13 | Code for T-cadherin involved in vascular homeostasis. [29] | Plasma membrane, extracellular |

Supporting References


[1] E. Holm, J. E. Aubin, G. K. Hunter, F. Beier, H. A. Goldberg, *Bone* **2015**, *71*, 145.

[2] S. Miura, J. Kondo, A. Takimoto, H. Sano-Takai, L. Guo, C. Shukunami, H. Tanaka, Y. Hiraki, *PLoS One* **2014**, *9*, 3.

[3] H. H. Lee, R. R. Behringer, *PLoS One* **2007**, *2*, 450.

[4] E. Koyama, E. B. Golden, T. Kirsch, S. L. Adams, R. A. S. Chandraratna, J. J. Michaille, M. Pacifici, *Dev. Biol.* **1999**, *208*, 375.

[5] A. Ionescu, E. Kozhemyakina, C. Nicolae, K. H. Kaestner, B. R. Olsen, A. B. Lassar, *Dev. Cell* **2012**, *22*, 927.

[6] T. N. Hissnauer, A. Baranowsky, J. M. Pestka, T. Streichert, K. Wiegandt, C. Goepfert, F. T. Beil, J. Albers, J. Schulze, P. Ueblacker, J. P. Petersen, T. Schinke, N. M. Meenen, R. Pörtner, M. Amling, *Osteoarthr. Cartil.* **2010**, *18*, 1630.

[7] V. Ulici, C. G. James, K. D. Hoenselaar, F. Beier, *PLoS One* **2010**, *5*, e8866.

[8] E. J. Mackie, Y. a Ahmed, L. Tatarczuch, K.-S. Chen, M. Mirams, *Int. J. Biochem. Cell Biol.* **2008**, *40*, 46.

[9] Y. Jing, J. Jing, L. Ye, X. Liu, S. E. Harris, R. J. Hinton, J. Q. Feng, *Sci. Rep.* **2017**, *7*, 10020.

[10] L. Ye, Y. Mishina, D. Chen, H. Huang, S. L. Dallas, M. R. Dallas, P. Sivakumar, T. Kunieda, T. W. Tsutsui, A. Boskey, L. F. Bonewald, J. Q. Feng, *J. Biol. Chem.* **2005**, *280*, 6197.

[11] D. Nurminsky, C. Magee, L. Faverman, M. Nurminskaya, *Dev. Biol.* **2007**, *302*, 427.

[12] H. Rosen, Z. Bar-Shavit, *J. Cell. Biochem.* **1994**, *55*, 334.

[13] T. Yasuda, K. Shimizu, Y. Nakagawa, S. Yamamoto, H. Niibayashi, T. Yamamuro, *Dev. Biol.* **1995**, *170*, 159.

[14] T. Saito, T. Ikeda, K. Nakamura, U. Il Chung, H. Kawaguchi, *EMBO Rep.* **2007**, *8*, 504.

[15] C. A. Yoshida, T. Kawane, T. Moriishi, A. Purushothaman, T. Miyazaki, H. Komori, M. Mori, X. Qin, A. Hashimoto, K. Sugahara, K. Yamana, K. Takada, T. Komori, *J. Biol. Chem.* **2014**, *289*, 26584.

[16] Y. Li, D. A. Lacerda, M. L. Warman, D. R. Beier, H. Yoshioka, Y. Ninomiya, J. T. Oxford, N. P. Morris, K. Andrikopoulos, F. Ramirez, B. B. Wardell, G. D. Lifferth, C. Teuscher, S. R. Woodward, B. A. Taylor, R. E. Seegmiller, B. R. Olsen, *Cell* **1995**, *80*, 423.

[17] C. C. Cheng, S. J. Chang, Y. N. Chueh, T. S. Huang, P. H. Huang, S. M. Cheng, T. N. Tsai, J. W. Chen, H. W. Wang, *BMC Genomics* **2013**, *14*, 182.

[18] C. Carbone, G. Piro, V. Merz, F. Simionato, R. Santoro, C. Zecchetto, G. Tortora, D. Melisi, *Int. J. Mol. Sci.* **2018**, *19*, 431.

[19] H. P. Gerber, T. H. Vu, A. M. Ryan, J. Kowalski, Z. Werb, N. Ferrara, *Nat. Med.* **1999**, *5*, 623.

[20] C. F. Tu, Y. T. Yan, S. Y. Wu, B. Djoko, M. T. Tsai, C. J. Cheng, R. B. Yang, *J. Biol. Chem.* **2008**, *283*, 12478.

[21] Y. Xiao, X. Wei, Z. Jiang, X. Wang, W. Ye, X. Liu, M. Zhang, Y. Xu, D. Wu, L. Lai, H. Yao, Z. Liu, S. Cao, P. Liu, B. Xu, Y. Li, Y. Yao, D. Pei, P. Li, *J. Hematol. Oncol.* **2015**, *8*, 7.

[22] E. F. Rocnik, P. Liu, K. Sato, K. Walsh, C. Vaziri, *J. Biol. Chem.* **2006**, *281*, 22855.

[23] S. Pirotte, V. Lamour, V. Lambert, M. L. A. Gonzalez, S. Ormenese, A. Noël, D. Mottet, V. Castronovo, A. Bellahcène, *Blood* **2011**, *117*, 2515.

[24] A. Rattner, H. Yu, J. Williams, P. M. Smallwood, J. Nathans, *Proc. Natl. Acad. Sci.* **2013**, *110*, E3830.

[25] S. Saadoun, M. C. Papadopoulos, M. Hara-Chikuma, A. S. Verkman, *Nature* **2005**, *434*, 786.

[26] E. W. L. Law, A. K. L. Cheung, V. I. Kashuba, T. V. Pavlova, E. R. Zabarovsky, H. L. Lung, Y. Cheng, D. Chua, D. Lai-Wan Kwong, S. W. Tsao, T. Sasaki, E. J. Stanbridge, M. L. Lung, *Oncogene* **2012**, *31*, 728.

[27] K. A. Uniewicz, M. J. Cross, D. G. Fernig, *J. Biol. Chem.* **2011**, *286*, 12.

[28] M. Tsujii, S. Kawano, S. Tsuji, H. Sawaoka, M. Hori, R. N. Dubois, *Cell* **1998**, *93*, 705.

[29] J. L. Parker-Duffen, K. Nakamura, M. Silver, R. Kikuchi, U. Tigges, S. Yoshida, M. S. Denzel, B. Ranscht, K. Walsh, *J. Biol. Chem.* **2013**, *288*, 24886.
